# Supplementary figures and images for: Hypermutator strains of Pseudomonas aeruginosa reveal novel pathways of resistance to combinations of cephalosporin antibiotics and beta-lactamase inhibitors
Source: PLoS Biol. 2022 Nov 18;20(11):e3001878. doi: 10.1371/journal.pbio.3001878 (PMC9718400; doi:10.1371/journal.pbio.3001878)

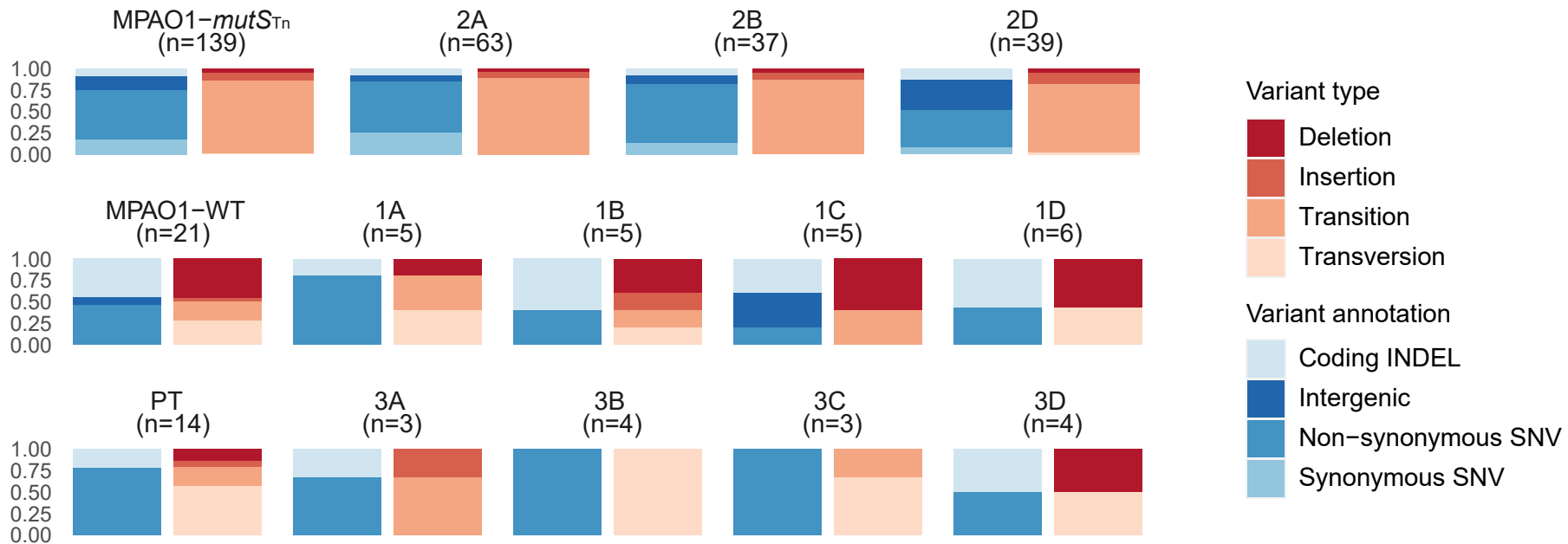

Supplement: S1 Fig — The total number of fixed variants acquired is indicated in parentheses. The underlying data to generate this figure can be found in S2 Data. (PDF) [file pbio.3001878.s001.pdf]

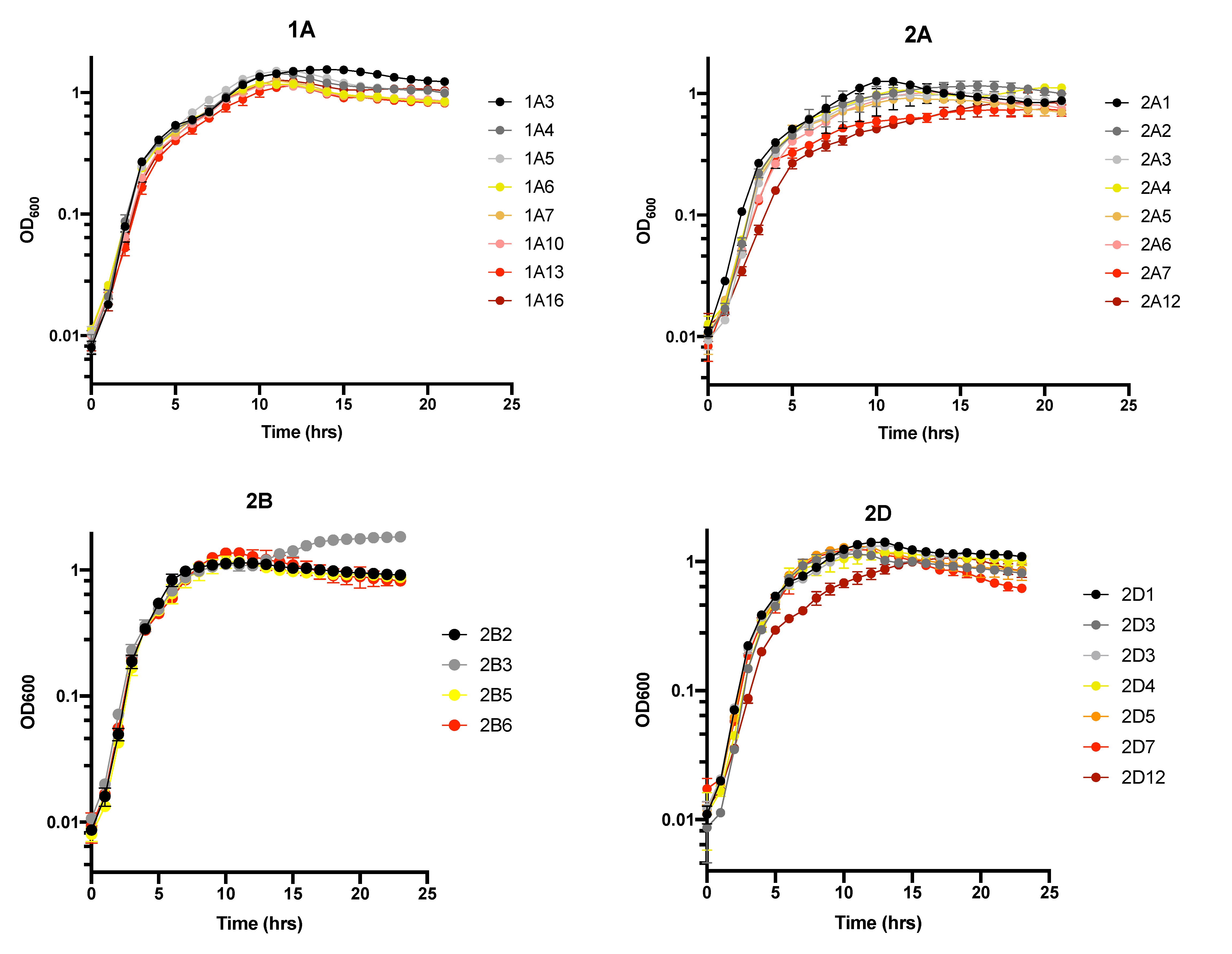

Supplement: S4 Fig — Growth curves of isolates from MPAO1-WT lineage 1A and MPAO1-mutSTn lineages 2A, 2B, and 2D (LB broth at 37°C). The passage number for each curve is indicated next to the lineage name in each legend. Experiments were repeated in triplicate, and time points represent the average OD600 with whiskers indicating the range. The underlying data to generate this figure can be found in S2 Data. (TIF) [file pbio.3001878.s004.tif]

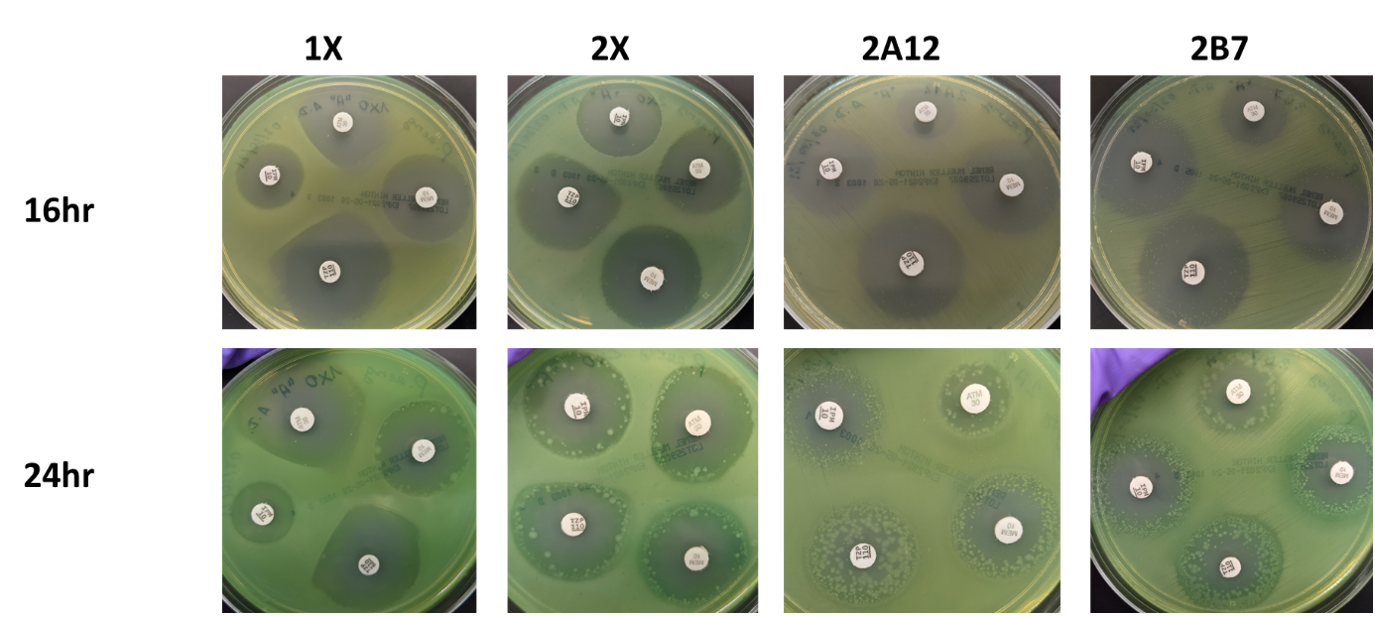

Supplement: S10 Fig — Representative photos of Kirby–Bauer susceptibility testing with ATM, IPM, MEM, and piperacillin-tazobactam (TZP) in the MPAO1-WT ancestor (1X), MPAO1-mutSTn ancestor (2X), and 2 CZA-resistant MPAO1-mutSTn isolates (lineage 2A passage 12 and lineage 2B passage 7). Pictures display readings at both 16 h, as recommended per CLSI guidelines, as well as at 24 h of incubation at 37°C. Scattered colonies can be observed inside some zones of inhibition at 16 h, particularly in the MPAO1-mutSTn isolates and more markedly in the evolved CZA-resistant isolates. ATM, aztreonam; IPM, imipenem; MEM, meropenem; TZP, piperacillin-tazobactam. (PNG) [file pbio.3001878.s010.png]
