# Supplementary material for: Hypermutator strains of Pseudomonas aeruginosa reveal novel pathways of resistance to combinations of cephalosporin antibiotics and beta-lactamase inhibitors
Source: PLoS Biol. 2022 Nov 18;20(11):e3001878. doi: 10.1371/journal.pbio.3001878 (PMC9718400; doi:10.1371/journal.pbio.3001878)
Supplement: S1 Table — Number of isolates and fixed variants analyzed per strain and general WGS sample statistics. The graphical representation of the fixed variants acquired by genotype described here is shown in the bar plots in Fig 1. The numbers in parentheses indicate the percentage out of all variants in the given genotype. (DOCX) [file pbio.3001878.s012.docx]

ST1 Table. General characteristics of WGS libraries and fixed variants. Number of isolates and fixed variants analyzed per strain and general WGS sample statistics. The graphical representation of the fixed variants acquired by genotype described here is shown in the bar plots in Figure 1. The numbers in parentheses indicate the percentage out of all variants in the given genotype.

|  | **All genotypes** | **MPAO1-*mutS*^Tn^** | **MPAO1-WT** | **PT** |
| --- | --- | --- | --- | --- |
| **Total Reads (M)**  median (IQR) | 3.7 (1.9, 6.4) | 4.2 (2.1, 6.8) | 3.6 (1.8, 6.2) | 4.3 (1.8, 6.7) |
| **Median Coverage**  median (IQR) | 51.0 (26.5, 78.5) | 67.5 (42.5, 82.2) | 40.0 (25.0, 78.0) | 54.0 (28.0, 73.0) |
| **Pct. Bases with at least 5X coverage (%)**    median (IQR) | 99.0 (97.1, 99.4) | 99.1 (98.1, 99.5) | 98.4 (96.8, 99.3) | 99.7 (98.1, 99.8) |
| **Total variants** | 171 (100) | 139 (100) | 21 (100) | 14 (100) |
| **Variant type** |  |  |  |  |
| SNV (%) | 142 (83%) | 120 (86.3%) | 11 (52.4%) | 11 (78.6%) |
| INS (%) | 14 (8.1%) | 12 (8.6%) | 1 (4.8%) | 1 (7.1%) |
| DEL (%) | 18 (10.5%) | 7 (5.0%) | 9 (42.9%) | 2 (14.3%) |
| **Variant characteristics** |  |  |  |  |
| Intergenic (%) | 25 (14.6%) | 23 (16.5%) | 2 (9.5%) | 0 (0.0%) |
| In-frame Insertion (%) | 2 (1.2%) | 0 (0.0%) | 1 (4.8%) | 1 (7.1%) |
| Frameshift Variant (%) | 21 (12.2%) | 13 (9.3%) | 7 (33.3%) | 1 (7.1%) |
| Synonymous SNV (%) | 24 (14%) | 24 (17.1%) | 0 (0.0%) | 0 (0.0%) |
| Missense SNV (%) | 95 (55.2%) | 74 (52.9%) | 10 (47.6%) | 11 (78.6%) |
| Start lost/Stop gained (%) | 5 (2.9%) | 5 (3.6%) | 0 (0.0%) | 0 (0.0%) |
| Large deletion (%) | 1 (0.6%) | 0 (0.0%) | 1 (4.8%) | 0 (0.0%) |
| **Transitions/Transversions** |  |  |  |  |
| Transitions | 127 (74.2%) | 119 (85.6%) | 5 (23.8%) | 3 (21.4%) |
| Transversions | 15 (8.8%) | 1 (0.8%) | 6 (28.6%) | 8 (57.1%) |
