# Supplementary material for: Hypermutator strains of Pseudomonas aeruginosa reveal novel pathways of resistance to combinations of cephalosporin antibiotics and beta-lactamase inhibitors
Source: PLoS Biol. 2022 Nov 18;20(11):e3001878. doi: 10.1371/journal.pbio.3001878 (PMC9718400; doi:10.1371/journal.pbio.3001878)
Supplement: S2 Table — (DOCX) [file pbio.3001878.s013.docx]

ST2 Table. General characteristics of isolate PT reference assembly

| Total length (bp) | 6926097 |
| --- | --- |
| Number of contigs | 96 |
| Number of contigs (>= 5000 bp) | 35 |
| N50 | 366744 |
| L50 | 8 |
| GC content (%) | 66.11 |
| Putative CDS as annotated by Prokka | 6380 |
