# Supplementary material for: Hypermutator strains of Pseudomonas aeruginosa reveal novel pathways of resistance to combinations of cephalosporin antibiotics and beta-lactamase inhibitors
Source: PLoS Biol. 2022 Nov 18;20(11):e3001878. doi: 10.1371/journal.pbio.3001878 (PMC9718400; doi:10.1371/journal.pbio.3001878)
Supplement: S3 Table — CZA MICs are expressed in μg/mL. NA = passage was not sequenced. (DOCX) [file pbio.3001878.s014.docx]

ST3 Table. Fixed variants acquired in the PT lineages. CZA MICs are expressed in µg/mL. NA = passage was not sequenced

|  |  |  |  |  | |  | |  | **PASSAGE** | | |  | | |  | |
| --- | --- | --- | --- | --- | --- | --- | --- | --- | --- | --- | --- | --- | --- | --- | --- | --- |
|  |  | **1** | | | **2** | | **3** | | | **4** | **5** | | **6** | **7** | | **12** |
| 3A | MIC | 4 | | | 4 | | 8 | | | 16 | 32 | | 32 | 32 | | 32 |
|  | Variants |  | | |  | | *nalD* H56R | | |  | *clpA* L606P | |  |  | | *phoQ* c.1087-1092 dupTGCCTG |
| 3B | MIC | 2 | | | 2 | | 8 | | | 32 | >256 | | >256 | >256 | | NA |
|  | Variants |  | | |  | |  | | | *pepA* 2 T17P | PDC c.710-730del (R238-L244del) | |  | *mpl* Y35S | |  |
| 3C | MIC | 2 | | | 8 | | 16 | | | 8 | 16 | | 16 | 128 | | NA |
|  | Variants |  | | | *mexR* L123P | |  | | |  | *clpA* Y545C | |  | *czcS* 3 (N47fs)  *clpA* L566S | |  |
| 3D | MIC | 2 | | | 4 | | 8 | | | 16 | 16 | | 32 | 128 | | 128 |
|  | Varints |  | | |  | |  | | | *nalD* L85Q | *clpA* c.1516delA (K506fs) | |  |  | | *phoQ* V260G  *mpl* p.Asp255-Glu260del |
