# Supplementary material for: Hypermutator strains of Pseudomonas aeruginosa reveal novel pathways of resistance to combinations of cephalosporin antibiotics and beta-lactamase inhibitors
Source: PLoS Biol. 2022 Nov 18;20(11):e3001878. doi: 10.1371/journal.pbio.3001878 (PMC9718400; doi:10.1371/journal.pbio.3001878)
Supplement: S4 Table — Three lineages (A, B, and C; left) passaged without CZA, compared to the 3 lineages passaged with CZA (2A, 2B, 2D; right), and also described in Fig 2. The numbering following the CZA lineage indicates the passage number in which the variant was identified, followed by the isolate number (e.g., 2A4-2 indicates lineage 2A, passage 4, isolate 2). A plus sign indicates presence of the variant. Coordinate positions are given with respect to the PAO1 reference chromosome. The MPAO1-WT lineages did not acquire any variants in shared targets. FS, frameshift mutation. (DOCX) [file pbio.3001878.s015.docx]

**ST4 Table. Variants in genes mutated in MPAO1-*mutS^Tn^* lineages that were passaged with and without CZA.** Three lineages (A, B and C, left) passaged without CZA, compared to the three lineages passaged with CZA (2A, 2B, 2D, right), and also described in Figure 2. The numbering following the CZA lineage indicates the passage number in which the variant was identified, followed by the isolate number (e.g. 2A4-2 indicates lineage 2A, passage 4, isolate 2). A plus sign indicates presence of the variant. Coordinate positions are given with respect to the PAO1 reference chromosome. The MPAO1-WT lineages did not acquire any variants in shared targets. FS = frameshift mutation.

|  |  |  |  |  | **Passaged without CZA** | | | **Passaged with CZA** | | | | | |
| --- | --- | --- | --- | --- | --- | --- | --- | --- | --- | --- | --- | --- | --- |
| **Gene** | **Position** | **Reference** | **Variant** | **Annotation** | **Lineage A** | **Lineage B** | **Lineage C** | **2A4-2** | **2A4-1** | **2B3-1** | **2B5-2** | **2B7-1** | **2D4-1** |
| *pdtA* | 758688 | C | T | P2911L | **+** | **+** | **+** |  |  |  |  |  |  |
| *pdtA* | 754530 | AGGGGGGG  CAGGC | AGGGGGGGG  CAGGC | FS |  |  |  |  | **+** |  |  |  |  |
| *pdtA* | 755576 | A | G | T1874A |  |  |  |  |  | **+** |  |  |  |
| *amaB* | 1114264 | AGGGGGGG  CGGG | AGGGGGGGG  CGGG | FS | + |  |  |  |  |  |  |  |  |
| *amaB* | 1114264 | AGGGGGGG  CGGGTGTT | AGGGGGGGG  CGGGTGTT | FS |  | + | + | + |  |  |  |  |  |
| PA2462 | 2767285 | GCCCCCC  GCAT | GCCCCCCC  GCAT | FS |  | **+** |  |  |  |  |  |  |  |
| PA2462 | 2764410 | G | A | Q4799* |  |  |  |  |  | **+** |  |  |  |
| PA2462 | 2766678 | A | G | Y4043H |  |  |  |  |  |  |  | **+** |  |
| *pilY1* | 5101022 | GCCCCC  AACT | GCCCC  AACT | FS |  |  | + |  |  |  |  |  |  |
| *pilY1* | 5102456 | C | T | Q592* |  |  |  |  |  |  |  |  | + |
| *rfaE* | 5612738 | G | A | R319C |  |  | + |  |  |  |  |  |  |
| *rfaE* | 5613314 | T | C | T127A |  |  |  |  |  |  | + |  |  |
