# Supplementary material for: Hypermutator strains of Pseudomonas aeruginosa reveal novel pathways of resistance to combinations of cephalosporin antibiotics and beta-lactamase inhibitors
Source: PLoS Biol. 2022 Nov 18;20(11):e3001878. doi: 10.1371/journal.pbio.3001878 (PMC9718400; doi:10.1371/journal.pbio.3001878)
Supplement: S5 Table — Five biological replicates (A–E) from each of the 3 mutants that underwent extended antibiotic testing by the Kirby–Bauer method as described. Results are given as zone diameters in mm and corresponding CLSI breakpoint susceptibility interpretations are given in parentheses. AMK, Amikacin; CI, Ciprofloxacin; GEN, Gentamycin; TOB, Tobramycin. (DOCX) [file pbio.3001878.s016.docx]

**ST5 Table. Extended susceptibility testing results for the engineered mutants.** Five biological replicates (A-E) from each of the three mutants that underwent extended antibiotic testing by the Kirby-Bauer method as described. Results are given as zone diameters in mm and corresponding CLSI breakpoint susceptibility interpretations are given in parentheses. AMK = Amikacin; TOB = Tobramycin; GEN = Gentamycin; CIP = Ciprofloxacin.

|  | MPAO1-WT | | | | | *mexV* -82:T>C | | | | | MexW E36K | | | | | *mexV* -82:T>C +  MexW E36K | | | | |
| --- | --- | --- | --- | --- | --- | --- | --- | --- | --- | --- | --- | --- | --- | --- | --- | --- | --- | --- | --- | --- |
| **Rep** | **A** | **B** | **C** | **D** | **E** | **A** | **B** | **C** | **D** | **E** | **A** | **B** | **C** | **D** | **E** | **A** | **B** | **C** | **D** | **E** |
| AMK | 25 (S) | 26 (S) | 24 (S) | 24 (S) | 25 (S) | 26 (S) | 25 (S) | 26 (S) | 26 (S) | 25 (S) | 25 (S) | 25 (S) | 26 (S) | 25 (S) | 24 (S) | 25 (S) | 24 (S) | 24 (S) | 26 (S) | 25 (S) |
| TOB | 24 (S) | 24 (S) | 24 (S) | 24 (S) | 23 (S) | 25 (S) | 24 (S) | 25 (S) | 24 (S) | 23 (S) | 24 (S) | 23 (S) | 23 (S) | 23 (S) | 23 (S) | 24 (S) | 23 (S) | 24 (S) | 24 (S) | 23 (S) |
| GEN | 23 (S) | 22 (S) | 22 (S) | 23 (S) | 20 (S) | 22 (S) | 22 (S) | 22 (S) | 21 (S) | 21 (S) | 21 (S) | 22 (S) | 22 (S) | 21 (S) | 21 (S) | 22 (S) | 21 (S) | 21 (S) | 21 (S) | 20 (S) |
| CIP | 24 (I) | 20 (I) | 20 (I) | 21 (I) | 23 (I) | 19 (I) | 21 (I) | 20 (I) | 23 (I) | 22 (I) | 18 (S) | 20 (I) | 19 (I) | 22 (I) | 21 (I) | 20 (I) | 18 (S) | 20 (I) | 20 (I) | 20 (I) |
