# Supplementary material for: Hypermutator strains of Pseudomonas aeruginosa reveal novel pathways of resistance to combinations of cephalosporin antibiotics and beta-lactamase inhibitors
Source: PLoS Biol. 2022 Nov 18;20(11):e3001878. doi: 10.1371/journal.pbio.3001878 (PMC9718400; doi:10.1371/journal.pbio.3001878)
Supplement: S6 Table — Susceptibility testing in the hypermutators was complicated by the appearance of satellite colonies within the inner zones. We made the assumption that the outer zone reflected resistance conferred by the genomic background of the hypermutator and that the inner zone satellite colonies represented mutants able to resist higher concentrations of antibiotic. In order to determine the outer zone diameter, we visually identified the zone of confluent bacterial growth and measured the corresponding diameter. We then measured the inner diameter by measuring the distance between the disk edge and the closest satellite colony and calculated the diameter using this distance. Diameters are shown in mm except for C/T, where testing was performed by E-test and results are show in μg/mL. Values within parentheses correspond to the CLSI interpretation of the zone diameter. NA = no satellite colonies seen within zone. Letter in parenthesis indicates biological replicate and R1 and R2 represent technical replicates. Pip-Tazo = Piperacillin-Tazobactam. (DOCX) [file pbio.3001878.s017.docx]

**ST6 Table: Antimicrobial susceptibility testing of MPAO1-*mutS^Tn^* ancestor and terminal isolates.** Susceptibility testing in the hypermutators was complicated by the appearance of satellite colonies within the inner zones. We made the assumption that the outer zone reflected resistance conferred by the genomic background of the hypermutator and that the inner zone satellite colonies represented mutants able to resist higher concentrations of antibiotic. In order to determine the outer zone diameter, we visually identified the zone of confluent bacterial growth and measured the corresponding diameter. We then measured the inner diameter by measuring the distance between the disk edge and the closest satellite colony, and calculated the diameter using this distance. Diameters are shown in mm except for C/T, where testing was performed by Etest and results are show in μg/mL. Values within parentheses correspond to the CLSI interpretation of the zone diameter. NA = no satellite colonies seen within zone. Letters in parentheses indicates biological replicate and R1 and R2 represent technical replicates. Pip-Tazo = Piperacillin-Tazobactam.

|  | **MPAO1-*mutS*^Tn^ ancestor** | | **Lineage 2A Passage 12** | | **Lineage 2B**  **Passage 7** | | **Lineage 2D**  **Passage 12** | |
| --- | --- | --- | --- | --- | --- | --- | --- | --- |
|  | **Outer** | **Inner** | **Outer** | **Inner** | **Outer** | **Inner** | **Outer** | **Inner** |
| **Pip-Tazo (A)** | 29 (S) | NA | 27 (S) | 10 (R) | 29 (S) | 20 (I) | 31 (S) | NA |
| **Pip-Tazo (B, R1)** | 27 (S) | NA | 26 (S) | 12 (R) | 31 (S) | 16 (I) | 29 (S) | NA |
| **Pip-Tazo (B, R2)** | 27 (S) | NA | 25 (S) | 12 (R) | 31 (S) | 16 (I) | 28 (S) | NA |
| **Meropenem (A)** | 22 (S) | NA | 25 (S) | 15 (R) | 28 (S) | 18 (I) | 29 (S) | 18 (I) |
| **Meropenem (B, R1)** | 30 (S) | NA | 27 (S) | 21 (S) | 25 (S) | 20 (S) | 29 (S) | NA |
| **Meropenem (B, R2)** | 31 (S) | NA | 28 (S) | 21 (S) | 27 (S) | 20 (S) | 28 (S) | NA |
| **Imipenem (A)** | 26 (S) | NA | 19 (S) | NA | 22 (S) | NA | 24 (S) | NA |
| **Imipenem (B, R1)** | 27 (S) | NA | 21 (S) | NA | 30 (S) | 20 (S) | 11 (R) | NA |
| **Imipenem (B, R2)** | 27 (S) | NA | 21 (S) | 18 (I) | 30 (S) | 21 (S) | 12 (R) | NA |
| **Aztreonam (A)** | 24 (S) | NA | 21 (S) | NA | 20 (S) | NA | 22 (S) | NA |
| **Aztreonam (B, R1)** | 26 (S) | NA | 17 (I) | NA | 18 (I) | NA | 13 (R) | NA |
| **Aztreonam (B, R2)** | - | - | 17 (I) | NA | 20 (I) | NA | 13 (R) | NA |
| **Cefepime** | 27 (S) | NA | 6 (R) | NA | 8 (R) | NA | 6 (R) | NA |
| **C/T (E-test)** | 1 (S) | NA | >256 (R) | NA | 32 (R) | NA | 64 (R) | NA |
