# Supplementary material for: Hypermutator strains of Pseudomonas aeruginosa reveal novel pathways of resistance to combinations of cephalosporin antibiotics and beta-lactamase inhibitors
Source: PLoS Biol. 2022 Nov 18;20(11):e3001878. doi: 10.1371/journal.pbio.3001878 (PMC9718400; doi:10.1371/journal.pbio.3001878)
Supplement: S7 Table — (DOCX) [file pbio.3001878.s018.docx]

**ST7 Table: P-values of two-sided Wilcoxon tests performed between the E-test MICs of engineered mutants (MPAO1** ***mexV* -82:T>C, MPAO1 MexW E36K and MPAO1 *mexV* -82:T>C + MexW E36K) and MPAO1-WT for the indicated agents.**

| COMP A | COMP B | CTX | CAZ | PIP | P/T | FEP | MEM | AZN | CZA | C/T |
| --- | --- | --- | --- | --- | --- | --- | --- | --- | --- | --- |
| *mexV* -82:T>C + MexW E36K | WT | 0.037 | 1.2 x 10^-4^ | 0.210 | 0.425 | 1.4 x 10^-4^ | 0.588 | 0.077 | 6.7 x 10^-5^ | 1.2 x 10^-4^ |
| *mexV* -82:T>C | WT | 0.423 | 1.4 x 10^-4^ | 0.840 | 0.068 | 0.006 | 0.938 | 0.334 | 2.4 x 10^-5^ | 1.0 x 10^-4^ |
| MexW E36K | WT | 0.204 | 0.003 | 0.567 | 0.224 | 1.0 x 10^-4^ | 1 | 0.697 | 0.001 | 0.002 |
| MexW E36K | *mexV* -82:T>C | 0.578 | 4.3 x 10^-4^ | 0.934 | 0.815 | 0.046 | 0.875 | 0.512 | 4.1 x 10^-5^ | 0.014 |
| *mexV* -82:T>C + MexW E36K | *mexV* -82:T>C | 0.110 | 1.9 x 10^-4^ | 0.369 | 0.507 | 1.4 x 10^-4^ | 0.477 | 0.047 | 4.8 x 10^-5^ | 1.0 x 10^-4^ |
| *mexV* -82:T>C + MexW E36K | MexW E36K | 0.292 | 1.4 x 10^-4^ | 0.305 | 0.640 | 1.3 x 10^-4^ | 0.636 | 0.079 | 1.0 x 10^-4^ | 1.3 x 10^-4^ |

COMP = comparator; CTX = cefotaxime; CAZ = ceftazidime; PIP = piperacillin, P/T = piperacillin/tazobactam; FEP = cefepime; MEM = meropenem; AZN = aztreonam; CZA = ceftazidime/avibactam; C/T = ceftolozane/tazobactam
