# Supplementary material for: Hypermutator strains of Pseudomonas aeruginosa reveal novel pathways of resistance to combinations of cephalosporin antibiotics and beta-lactamase inhibitors
Source: PLoS Biol. 2022 Nov 18;20(11):e3001878. doi: 10.1371/journal.pbio.3001878 (PMC9718400; doi:10.1371/journal.pbio.3001878)
Supplement: S8 Table — Orthologous regions neighboring 2 mutations in the MexVW operon were identified in a set of 7,493 P. aeruginosa genomes from the NCBI Pathogen Detection database. (a) Summary of the nucleotide sequence alignment in proximity to PA4373-mexV +/- 10 bases. (b) Amino acid sequence surrounding MexW E36K with the corresponding residue highlighted in orange background. (DOCX) [file pbio.3001878.s019.docx]

**ST8 Table. Consensus sequences and nucleotide and amino-acid frequency tables in proximity to PA4373-mexV (4903384 T>C) and MexW E36K variants in 7493 genomes from the NCBI pathogen database.** Orthologous regions neighboring two mutations in the MexVW operon were identified in a set of 7493 *P. aeruginosa* genomes from the NCBI Pathogen detection database. (**A**) Summary of the nucleotide sequence alignment in proximity to PA4373-*mexV* +/- 10 bases. (**B**) Amino acid sequence surrounding MexW E36K with the corresponding residue highlighted in orange background.

**A. PA4373-*mexV* (4903384 T🡪C)**

|  | A | T | C | C | T | G | T | G | C | T | T | T | G | A | C | A | A | G |
| --- | --- | --- | --- | --- | --- | --- | --- | --- | --- | --- | --- | --- | --- | --- | --- | --- | --- | --- |
| A | 7417 | 0 | 0 | 0 | 0 | 0 | 0 | 0 | 0 | 0 | 0 | 0 | 0 | 7435 | 0 | 7435 | 7435 | 2 |
| C | 0 | 38 | 7434 | 7435 | 0 | 18 | 0 | 0 | 7435 | 0 | 0 | 0 | 0 | 0 | 7434 | 0 | 0 | 0 |
| G | 18 | 0 | 0 | 0 | 23 | 7417 | 0 | 7435 | 0 | 0 | 0 | 0 | 7435 | 0 | 0 | 0 | 0 | 7433 |
| T | 0 | 7397 | 1 | 0 | 7412 | 0 | 7435 | 0 | 0 | 7435 | 7435 | 7435 | 0 | 0 | 1 | 0 | 0 | 0 |
| - | 2 | 2 | 2 | 2 | 2 | 2 | 2 | 2 | 2 | 2 | 2 | 2 | 2 | 2 | 2 | 2 | 2 | 2 |

**B. MexW E36K**

|  | **Q** | **A** | **F** | **S** | **K** | **L** | **V** | **I** | **R** | **E** | **Y** | **P** | **Q** | **M** | **E** | **N** | **A** | **L** |
| --- | --- | --- | --- | --- | --- | --- | --- | --- | --- | --- | --- | --- | --- | --- | --- | --- | --- | --- |
| - | 132 | 132 | 132 | 132 | 132 | 132 | 132 | 132 | 132 | 132 | 132 | 132 | 132 | 132 | 132 | 132 | 132 | 132 |
| A | 0 | 7384 | 0 | 0 | 0 | 0 | 0 | 0 | 0 | 0 | 0 | 0 | 0 | 0 | 0 | 0 | 7383 | 0 |
| E | 0 | 0 | 0 | 0 | 0 | 0 | 0 | 0 | 0 | 7384 | 0 | 0 | 0 | 0 | 7384 | 0 | 0 | 0 |
| F | 0 | 0 | 7384 | 0 | 0 | 0 | 0 | 0 | 0 | 0 | 0 | 0 | 0 | 0 | 0 | 0 | 0 | 0 |
| G | 0 | 0 | 0 | 0 | 0 | 0 | 0 | 0 | 0 | 0 | 0 | 0 | 0 | 0 | 0 | 0 | 0 | 0 |
| I | 0 | 0 | 0 | 0 | 0 | 0 | 0 | 7384 | 0 | 0 | 0 | 0 | 0 | 0 | 0 | 0 | 0 | 0 |
| K | 0 | 0 | 0 | 0 | 7384 | 0 | 0 | 0 | 0 | 0 | 0 | 0 | 0 | 0 | 0 | 0 | 0 | 0 |
| L | 0 | 0 | 0 | 0 | 0 | 7384 | 0 | 0 | 0 | 0 | 0 | 2 | 0 | 0 | 0 | 0 | 0 | 7384 |
| M | 0 | 0 | 0 | 0 | 0 | 0 | 7 | 0 | 0 | 0 | 0 | 0 | 0 | 7384 | 0 | 0 | 0 | 0 |
| N | 0 | 0 | 0 | 0 | 0 | 0 | 0 | 0 | 0 | 0 | 0 | 0 | 0 | 0 | 0 | 7384 | 0 | 0 |
| P | 0 | 0 | 0 | 0 | 0 | 0 | 0 | 0 | 0 | 0 | 0 | 7382 | 0 | 0 | 0 | 0 | 0 | 0 |
| Q | 7384 | 0 | 0 | 0 | 0 | 0 | 0 | 0 | 0 | 0 | 0 | 0 | 7384 | 0 | 0 | 0 | 0 | 0 |
| R | 0 | 0 | 0 | 0 | 0 | 0 | 0 | 0 | 7383 | 0 | 0 | 0 | 0 | 0 | 0 | 0 | 0 | 0 |
| S | 0 | 0 | 0 | 7384 | 0 | 0 | 0 | 0 | 1 | 0 | 0 | 0 | 0 | 0 | 0 | 0 | 0 | 0 |
| T | 0 | 0 | 0 | 0 | 0 | 0 | 0 | 0 | 0 | 0 | 0 | 0 | 0 | 0 | 0 | 0 | 0 | 0 |
| V | 0 | 0 | 0 | 0 | 0 | 0 | 7377 | 0 | 0 | 0 | 0 | 0 | 0 | 0 | 0 | 0 | 1 | 0 |
| X | 0 | 0 | 0 | 0 | 0 | 0 | 0 | 0 | 0 | 0 | 0 | 0 | 0 | 0 | 0 | 0 | 0 | 0 |
| Y | 0 | 0 | 0 | 0 | 0 | 0 | 0 | 0 | 0 | 0 | 7384 | 0 | 0 | 0 | 0 | 0 | 0 | 0 |
