# Supplementary material for: Hypermutator strains of Pseudomonas aeruginosa reveal novel pathways of resistance to combinations of cephalosporin antibiotics and beta-lactamase inhibitors
Source: PLoS Biol. 2022 Nov 18;20(11):e3001878. doi: 10.1371/journal.pbio.3001878 (PMC9718400; doi:10.1371/journal.pbio.3001878)
Supplement: S9 Table — Percentage calculations in “Number of assemblies” column represent the number of assemblies containing variants in the given gene divided by the number of assemblies with at least 1 high-similarity BLASTp match for all proteins in the DNA repair system (n = 6,805). The percentage calculations in “Number of variants” column represent the number of unique variants divided by the same denominator above. This latter percentage represents a conservative estimate of the variant frequency adjusted for clonality with the assumption that all assemblies containing a given variant are clonal. (DOCX) [file pbio.3001878.s020.docx]

**ST9 Table. Highly disruptive variants in MMR and BER proteins in the NCBI Pathogen Detection Database.** Percentage calculations in “Number of assemblies” column represent the number of assemblies containing variants in the given gene divided by the number of assemblies with at least one high-similarity BLASTp match for all proteins in the DNA repair system (n= 6805). The percentage calculations in “Number of variants” column represent the number of unique variants divided by the same denominator above. This latter percentage represents a conservative estimate of the variant frequency adjusted for clonality with the assumption that all assemblies containing a given variant are clonal.

| **Gene** | **Number of variants (%)** | **Number of assemblies (%)** |
| --- | --- | --- |
| ***mutS*** | 77 (1.1) | 117 (1.7) |
| ***mutL*** | 53 (0.8) | 108 (1.6) |
| ***uvrD*** | 4 (0.06) | 5 (0.1) |
| ***mutT*** | 7 (0.1) | 20 (0.3) |
| ***mutY*** | 11 (0.16) | 15 (0.2) |
| ***mutM*** | 3 (0.04) | 8 (0.1) |
| **MMR or BER gene (total)** | 155 (2.3) | 260 (3.8) |
