# Supplementary material for: Hypermutator strains of Pseudomonas aeruginosa reveal novel pathways of resistance to combinations of cephalosporin antibiotics and beta-lactamase inhibitors
Source: PLoS Biol. 2022 Nov 18;20(11):e3001878. doi: 10.1371/journal.pbio.3001878 (PMC9718400; doi:10.1371/journal.pbio.3001878)
Supplement: S10 Table — Percentage calculations in the “Number of assemblies” column represent the number of assemblies containing variants in the given gene divided by the number of assemblies with at least 1 high-similarity BLASTp match for all proteins in the system (n = 6,820). The percentage calculations in “Number of variants” column represent the number of unique variants divided by the same denominator above. This latter percentage represents a conservative estimate of the variant frequency adjusted for clonality with the assumption that all assemblies containing a given variant are clonal. (DOCX) [file pbio.3001878.s021.docx]

ST10 Table: Highly disruptive variants in MexAB-OpM in the NCBI Pathogen Detection Database. Percentage calculations in the “Number of assemblies” column represent the number of assemblies containing variants in the given gene divided by the number of assemblies with at least one high-similarity BLASTp match for all proteins in the system (n = 6820). The percentage calculations in “Number of variants” column represent the number of unique variants divided by the same denominator above. This latter percentage represents a conservative estimate of the variant frequency adjusted for clonality with the assumption that all assemblies containing a given variant are clonal.

| **Protein** | **Number of variants (%)** | **Number of assemblies (%)** |
| --- | --- | --- |
| **MexA** | 86 (1.3) | 199 (2.9) |
| **MexB** | 148 (2.2) | 335 (4.9) |
| **OprM** | 33 (0.5) | 124 (1.8) |
| **MexAB-OprM (total)** | 267 (3.9) | 629 (9.2) |
