# Supplementary material for: Hypermutator strains of Pseudomonas aeruginosa reveal novel pathways of resistance to combinations of cephalosporin antibiotics and beta-lactamase inhibitors
Source: PLoS Biol. 2022 Nov 18;20(11):e3001878. doi: 10.1371/journal.pbio.3001878 (PMC9718400; doi:10.1371/journal.pbio.3001878)
Supplement: S11 Table — Passages indicated as “N/A” were not utilized for RNA-Seq analysis. (DOCX) [file pbio.3001878.s022.docx]

**ST11 Table. Passages from which terminal isolates were derived, for WGS and RNA-Seq analyses.** Passages indicated as “N/A” were not utilized for RNA-Seq analysis

| LINEAGE | WGS terminal passage | RNA-Seq terminal passage |
| --- | --- | --- |
| 1A | 16 | 15 |
| 1B | 20 | N/A |
| 1C | 20 | 14 |
| 1D | 20 | 16 |
| 2A | 12 | 7 |
| 2B | 7 | 6 |
| 2D | 12 | 7 |
| 3A | 12 | N/A |
| 3B | 7 | N/A |
| 3C | 7 | N/A |
| 3D | 12 | N/A |
