# Supplementary material for: Hypermutator strains of Pseudomonas aeruginosa reveal novel pathways of resistance to combinations of cephalosporin antibiotics and beta-lactamase inhibitors
Source: PLoS Biol. 2022 Nov 18;20(11):e3001878. doi: 10.1371/journal.pbio.3001878 (PMC9718400; doi:10.1371/journal.pbio.3001878)
Supplement: S12 Table — (DOCX) [file pbio.3001878.s023.docx]

**ST12 Table. Bacterial strains and plasmids used in this work**

|  | Relevant genotype or characteristics | Reference or Source |
| --- | --- | --- |
| Strains |  |  |
| *P. aeruginosa* MPAO1 | Wild type MPAO1 | PA Two-Allele Library University of Washington, Seattle, US[1-2] |
| MPAO1-*mutS*^Tn^ | PW7149 mutS-D02:: ISlacZ/hah | PA Two-Allele Library University of Washington, Seattle, US[1-2] |
| *P. aeruginosa* PT | Patient isolate comparator (blood) | [3] |
| NEB 5-alpha Competent *E. coli* | huA2 Δ(argF-lacZ)U169 phoA glnV44 Φ80 Δ(lacZ)M15 gyrA96 recA1 relA1 endA1 thi-1 hsdR17 | NEB (catalog number C2987H) |
| *E. coli* S17.1 (λ pir+) | Donor *E. coli* strain used in conjugational transfer of allelic exchange vector pEX18Gm into the *P. aeruginosa* MPA01 | Joe J. Harrison, Department of Biological Sciences, University of Calgary, Calgary, Alberta, Canada[4] |
|  |  |  |
| Plasmids |  |  |
| pEX18Gm | Allelic exchange vector /suicide cloning vector, GmR | Joe J. Harrison (see above) |
| pEX18Gm :: position 4903384 T – C | Genomic region with T>C mutation at position 4903384 cloned into allelic exchange vector pEX18Gm, GmR | This study |
| pEX18Gm :: *mexW* E36K | Genomic region with *mexW* (4904752 G>) mutation (yielding the MexW E36K substitution) cloned into allelic exchange vector pEX18Gm, GmR | This study |
| pEX18Gm :: *mexB* W753R | Genomic region with *mexB* (475447 T>C) mutation yielding the MexB W753R substitution) cloned into allelic exchange vector pEX18Gm, Gm^R^ | This study |
|  |  |  |
| Primers |  |  |
| M13F | Universal primer used to verify the insertion of mutant allele into allelic exchange vector Forward GTAAAACGACGGCCAG | Universal sequencing primer |
| M13R | Universal primer used to verify the insertion of mutant allele into allelic exchange vector CAGGAAACAGCTATGAC | Universal sequencing primer |
| *mexV*-F | To screen the colonies for T>C mutation at position 4903384  TTCATCTGGGACCTGGACAG | This study |
| *mexV*-R | To screen the colonies for T>C mutation at position 4903384  TTTTCGAGATGGCCTTGCTG | This study |
| *mexW* E36K -F | To screen the colonies for *mexW* E36K mutation GCGATCACCTTCACCCTCTA | This study |
| *mexW* E-36K-R | To screen the colonies for *mexW* E36K mutation  GTTGTTCATCTGCTCGCTGT | This study |
| *mexB* W753R-F | To screen the colonies for *mexB* W753R mutation  GCGGCCAGAGTTCGGGCAT | This study |
| *mexB* W753R -R | To screen the colonies for *mexB* W753R mutation  CAGAGGAACACCACCAGCAG | This study |

**REFERENCES**

1. Held K, Ramage E, Jacobs M, Gallagher L, Manoil C. Sequence-verified two-allele transposon mutant library for Pseudomonas aeruginosa PAO1. J Bacteriol. 2012;194(23):6387-9. Epub 2012/09/18. doi: 10.1128/JB.01479-12. PubMed PMID: 22984262; PubMed Central PMCID: PMCPMC3497512.

2. Jacobs MA, Alwood A, Thaipisuttikul I, Spencer D, Haugen E, Ernst S, et al. Comprehensive transposon mutant library of Pseudomonas aeruginosa. Proc Natl Acad Sci U S A. 2003;100(24):14339-44. Epub 2003/11/18. doi: 10.1073/pnas.2036282100. PubMed PMID: 14617778; PubMed Central PMCID: PMCPMC283593.

3. Khil PP, Dulanto Chiang A, Ho J, Youn JH, Lemon JK, Gea-Banacloche J, et al. Dynamic Emergence of Mismatch Repair Deficiency Facilitates Rapid Evolution of Ceftazidime-Avibactam Resistance in Pseudomonas aeruginosa Acute Infection. mBio. 2019;10(5). Epub 2019/09/19. doi: 10.1128/mBio.01822-19. PubMed PMID: 31530672; PubMed Central PMCID: PMCPMC6751058.

4. Hmelo LR, Borlee BR, Almblad H, Love ME, Randall TE, Tseng BS, et al. Precision-engineering the Pseudomonas aeruginosa genome with two-step allelic exchange. Nat Protoc. 2015;10(11):1820-41. Epub 2015/10/23. doi: 10.1038/nprot.2015.115. PubMed PMID: 26492139; PubMed Central PMCID: PMCPMC4862005.
