# Supplementary material for: Hypermutator strains of Pseudomonas aeruginosa reveal novel pathways of resistance to combinations of cephalosporin antibiotics and beta-lactamase inhibitors
Source: PLoS Biol. 2022 Nov 18;20(11):e3001878. doi: 10.1371/journal.pbio.3001878 (PMC9718400; doi:10.1371/journal.pbio.3001878)
Supplement: Attachment — Submitted filename: PLOS_BIOL_RTR_080722.pdf [file pbio.3001878.s030.pdf]

Response to Reviewers

Novel Mechanisms of Resistance to Ceftazidime/Avibactam and Ceftolozane/Tazobactam in Mismatch Repair-Deficient *Pseudomonas aeruginosa*

Dulanto Chiang A, Patil PP, Beka L, Youn JH, Launay A, Bonomo RA, Khil PP, Dekker JP

Manuscript # PBIOLOGY-D-22-00962R1

Editor's comments:

As you will see below, the reviewers find your work interesting, important and well executed, and appreciate that the paper is clearly written. However, they also raised several concerns that should be addressed before further consideration at PLOS Biology. In particular, we think it is important to address the concern of reviewer #2 that the main results may be anecdotal and hard to generalize. We consider that you should address all the reviewer comments, especially those regarding effects of mutation rate and population size.

Response: We sincerely thank the three reviewers and the Editor for the extremely thorough and thoughtful reviews of our manuscript. These critical comments and suggestions helped us to clarify many issues and have substantially improved the manuscript, and we are very highly appreciative of the effort and significant time to critique our work. Below we answer each of the questions raised. In particular, we have paid attention to addressing the issues of whether the MexAB-OprM inactivation we observed is an "anecdotal" or chance occurrence and the comments on starting inoculum, mutation rate and population size.

Reviewer #1 (Remarks to the Author)

In this manuscript, Chiang et al. study the mechanisms of resistance that develops to CZA in Pseudomonas that have hypermutation due to mismatch repair deficiencies. CZA is an important expanded spectrum antibiotic that has the beta-lactamase avibactam which inhibits AmpC, an important resistance mechanism in Pseudomonas. The authors demonstrate that MMR-deficient Pseudomonas evolves resistance through mechanisms that are not seen in WT lineages. This includes the finding that mutations in MexVW increase resistance to CZA which was found in evolved strains and then with mutations engineered into a WT background. This alternative resistance mechanism is certainly underappreciated and is an important finding. The paper is clear, thorough and well written.

Response: We greatly appreciate this reviewer's very careful and deep review of our manuscript and positive evaluation of our work.

A few points to be improved/clarified upon include:

1. *In the search for mutations in MexV and MexW it was noted that the mutations in this study were not found in sequenced isolates in public databases. Can the authors discuss the number of isolates in the database that had mutS mutations present? It would be helpful to know what subset of available genomes are thought to be deficient in MMR.*

<u>Response</u>: We thank the reviewer for raising this important question. Indeed, one would like to know exactly how many of the isolates in the public database we examined are hypermutators, either due to MutS deficiencies or other mechanisms. The definitive way to answer this question is experimentally, by testing mutational rates *in vitro*, given that it is difficult to predict the effects of uncharacterized variants in the *mutS* or other DNA repair genes on spontaneous mutation rates. Given that we did not have access to the isolates, we attempted to answer this question bioinformatically and attempted to identify genomes with altered and inactivated MutS proteins, as well as the MutL and UvrD components of the *P. aeruginosa* MMR system, and MutT, MutY and MutM components of the base excision repair (BER) system within the NCBI Pathogen Detection Database genome assemblies (n = 7492, date accessed: 05/14/2021).

We excluded assemblies with incomplete metadata or fragmented assemblies having >500 contigs (n = 615) to ensure quality of the analysis set. Then, using a BLASTp alignment we identified homologs of MutS, MutL, UvrD, MutT, MutY and MutM proteins (Table RR1, e-value $<10^{-4}$ and >94% identity to the PAO1 reference) in this refined set of genome assemblies.

Table RR1. DNA repair components BLASTp matches in the NCBI pathogen database (n assemblies = 6877). The respective reference PAO1 protein sequence was queried against each assembly in the NCBI pathogen detection database. A cutoff of >94% sequence identity and e-value of $<10^{-4}$ were used.

#BLASTp hits	MutS	MutL	UvrD	MutT	MutY	MutM
0	10	6	10	17	40	11
1	6758	6792	6865	6853	6834	6865
2	109	79	2	7	3	1
3	0	0	0	0	0	0
Percent >0	99.9%	99.9%	99.9%	99.8%	99.4%	99.8%

In assemblies with more than one BLASTp hit, we analyzed the lengths of homologous proteins to evaluate the possibility of having partial or full-length gene duplication (Figure RR1). We found that most detected homologous proteins with more than one blast hit were shorter than full length sequence of PAO1 reference, resulting from frameshifts and nonsense mutations found within CDSs. Nevertheless, three assemblies with apparent full-length DNA repair gene duplications were identified this way (see RR Methods) and were excluded from variant analysis. Coverage statistics were not provided for these assemblies, so our analysis comes with the caveat that other possible duplications may exist that were collapsed during assembly and cannot be excluded. Another caveat is the difficulty of controlling for assembly and sequencing artifacts and errors.

Next, we analyzed sequence variants in the remaining 6805 assemblies with at least one BLASTp match for the six DNA repair proteins, without evidence of partial or full-length duplications. We used snippy[1] to align these assembly contig sequences against the PAO1 reference genome by generating synthetic reads (snippy –contigs option), followed by snpEff[2] annotation and subsequent analysis of sequence variants in the MMR genes MutS, MutL, UvrD and the BER pathway genes MutT, MutY and MutM.

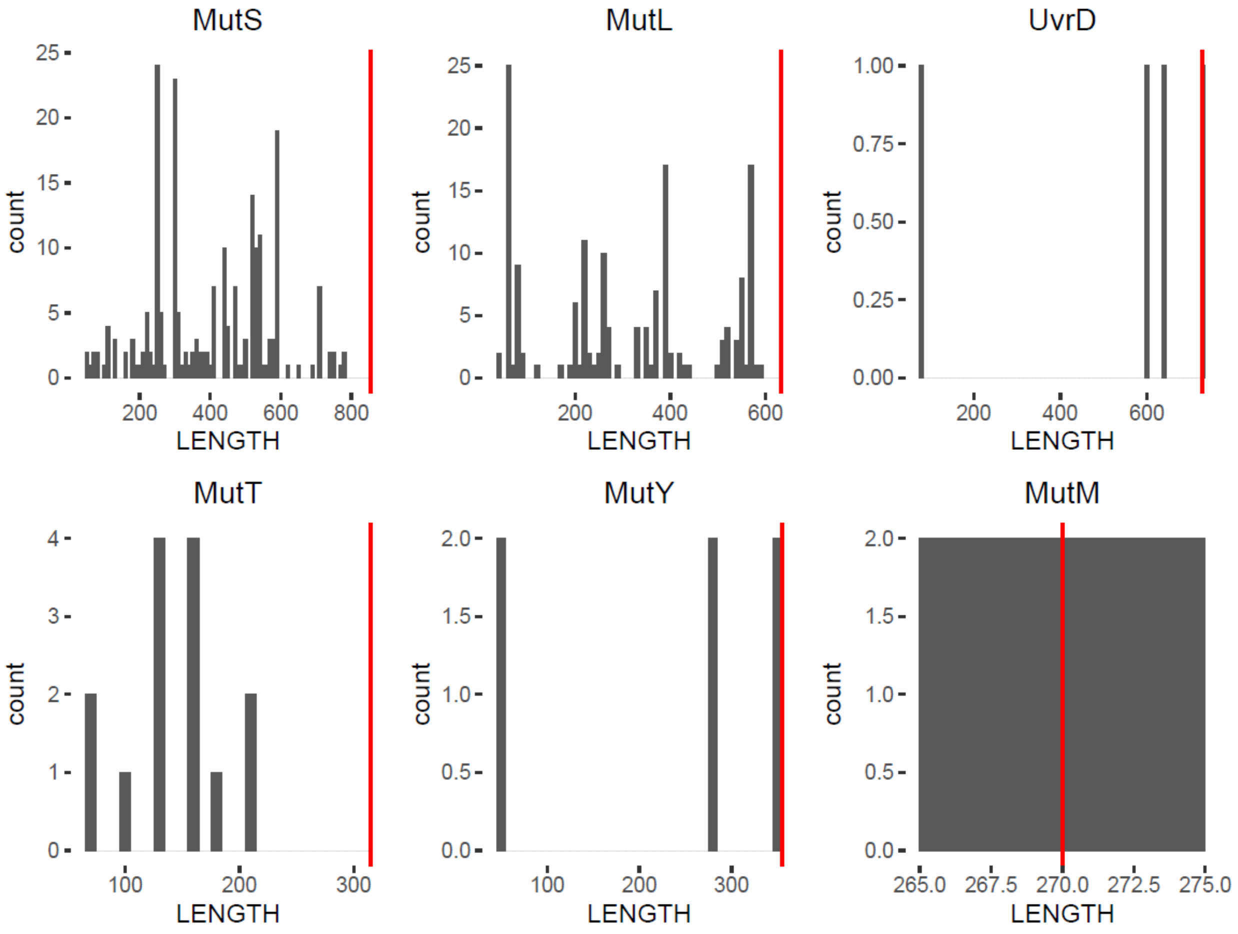

Figure RR1. BLASTp hit lengths in assemblies with >1 annotated hit for queried MMR and BER proteins. A red vertical line represents the full protein length as annotated in the PAO1 reference sequence. Assemblies with hits longer than 95% of the full protein length were individually interrogated for gene duplication.

We observed a total of 1073 variants coding for non-synonymous substitutions in *mutS*, *mutL*, and *uvrD*, genes in the MMR system (Table RR2). Out of these, 134 were frameshifting/stop gained/start lost variants predicted to inactivate key components of the MMR system and distributed among 226/6805 of assemblies (3.3%). To adjust for potential redundancy in this subset of assemblies, we conservatively assumed each individual disruptive variant (n = 134) to represent a single clonal lineage, obtaining an estimate of 134/6805 (1.97%) of independent mutations. We regard this 1.97% as an approximate lower boundary estimate, as it does not account for inactivating nonsynonymous variants. The total number of assemblies containing any nonsynonymous variant in the MMR components relative to the PAO1 was 5491/6805 (80.7%), so it is possible the fraction of isolates with actual MMR functional alterations may be substantially higher than 1.97%.

Table RR2. Sequence variant characteristics in DNA repair proteins among 6805 assemblies.

Type	MMR			BER			TOTAL MMR	TOTAL
	MutS	MutL	UvrD	MutT	MutY	MutM		
**Frameshift variant**	63	37	2	5	10	2	102	119
**Start lost / Stop gained**	14	16	2	2	1	1	32	36
**Missense variant**	362	329	220	179	178	120	911	1388
**Conservative inframe insertion/deletion**	7	5	0	0	1	0	12	13
**Disruptive inframe insertion/ deletion**	7	9	0	0	0	1	16	17
**Synonymous variant**	392	387	256	162	174	123	1035	1494
**TOTAL**	845	783	480	348	364	247	2108	3067
**TOTAL NON-SYN**	453	396	224	186	190	124	1073	1573
**TOTAL HIGH IMPACT**	77	53	4	7	11	3	134	155

Using similar methods, we identified 21 unique frameshifting/stop gained/start lost variants in *mutM, mutT* and *mutY* of the BER pathway, distributed throughout the coding sequences, and likely to be disruptive (Figure RR2).

Figure RR2. Distribution of stop gained/start lost/frameshifting variants along amino acid sequences of DNA repair proteins. The y-axis represents the aggregated sequence variant count, and the blue bar along the x-axis represents the full protein length as annotated in the PAO1 reference genome.

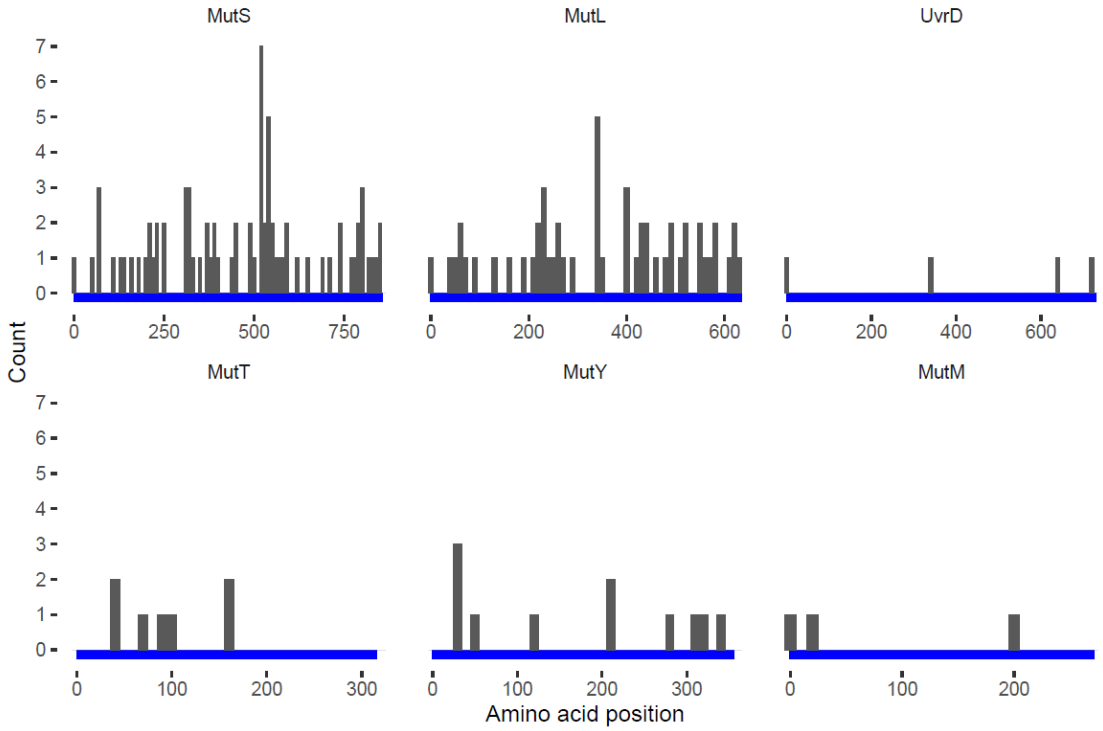

Taking both MMR and BER frameshifting/stop gained/start lost variants, the database contained 260/6805 (3.8%) isolates with at least one predicted inactivating mutation in *mutS, mutL, uvrD, mutT, mutY* or *mutM.* Once again, adjusting for clonality by assuming each unique high-impact variant to represent a single clonal lineage, a conservative estimate for putative hypermutators in this dataset would be 155/6805 (2.3%), which places what we believe is an approximate lower boundary estimate on the number of MMR and BER hypermutators (Table RR3, now included as Supplementary Table 9 in manuscript).

Table RR3. Percentage calculations in "Number of assemblies" column represent the number of assemblies containing variants in the given gene divided by the number of assemblies with at least one high-similarity BLASTp match for all proteins in the DNA repair system (n= 6805). The percentage calculations in "Number of variants" column represent the number of unique variants divided by the same denominator above. This latter percentage represents a conservative estimate of the variant frequency adjusted for clonality with the assumption that all assemblies containing a given variant are clonal.

	**Number of variants (%)**	**Number of assemblies (%)**
mutS	77 (1.1)	117 (1.7)
mutL	53 (0.8)	108 (1.6)
uvrD	4 (0.06)	5 (0.1)
mutT	7 (0.1)	20 (0.3)
mutY	11 (0.16)	15 (0.2)
mutM	3 (0.04)	8 (0.1)
**Total**	155 (2.3)	260 (3.8)

Again, these number do not include contributions of any of the identified nonsynonymous variants. A total of 6663/6805 (97.9%) of assemblies present at least one nonsynonymous variant in *mutS, mutL, uvrD, mutT, mutY* or *mutM*, compared the PAO1, making calculating an accurate upper bound of hypermutators challenging.

Of note, even though 81.2% of these 260 putative DNA repair-deficient isolates were of clinical origin, only 4 (1.8%) were assigned to a SNP cluster in the NCBI pathogen database (https://www.ncbi.nlm.nih.gov/pathogens/pathogens_help/#sequence-data). This is in contrast to 66.7% of the remaining clinical isolates in the database that were assigned to a SNP cluster. This indicates a greater degree of divergence among the putative hypermutators compared with other isolates, consistent with a hypermutator phenotype which would act to increase genomic distances due to accumulated mutations.

This information has been added to the manuscript as Supplemental Table 9 and as clarifying text in the main manuscript in lines 446-480, 634-638, and 994-1014 (tracked version).

2. *In addition, the authors note an early inactivating MexB mutation W735R. It would likewise be interesting to know how common the mutation is seen. If present in other genomes, how common was it?*

Response: We thank the reviewer for this important question which we have now clarified in the manuscript. Below we answer the specific question of how often the MexB W753R mutation occurs and attempt to answer the more general (and essential) question of how often inactivation of the MexAB-OprM efflux pump occurs through any mechanism, as this latter proportion would be the relevant one for our study. To do this, we utilized a similar variant calling approach as mentioned in the previous response to identify potential inactivating mutations in NCBI pathogen database assemblies that contained a BLASTp hit to all components of MexAB-OprM (n=6820, Table RR4) aligned against PAO1 reference.

Table RR4. MexAB-OprM protein BLASTp matches in the NCBI pathogen database (n assemblies = 6877). The respective reference PAO1 protein sequence was queried against each assembly in the NCBI pathogen detection database. A cutoff of >94% sequence identity and e-value of $<10^{-4}$ was used.

#BLASTp hits	MexA	MexB	OprM
0	16	19	43
1	6713	6626	6812
2	148	230	22
3	0	2	0
Percent >0	99.8%	99.7%	99.4%

We identified and excluded one assembly with a possible MexA duplication (Figure RR3) with the same methods as described above.

Figure RR3. BLASTp hit lengths in assemblies with >1 annotated hit for queried MexAB-OprM components: A red vertical line represents the full protein length as annotated in the PAO1 reference sequence. One assembly with two hits longer than 95% of the full MexA length was individually interrogated for gene duplication.

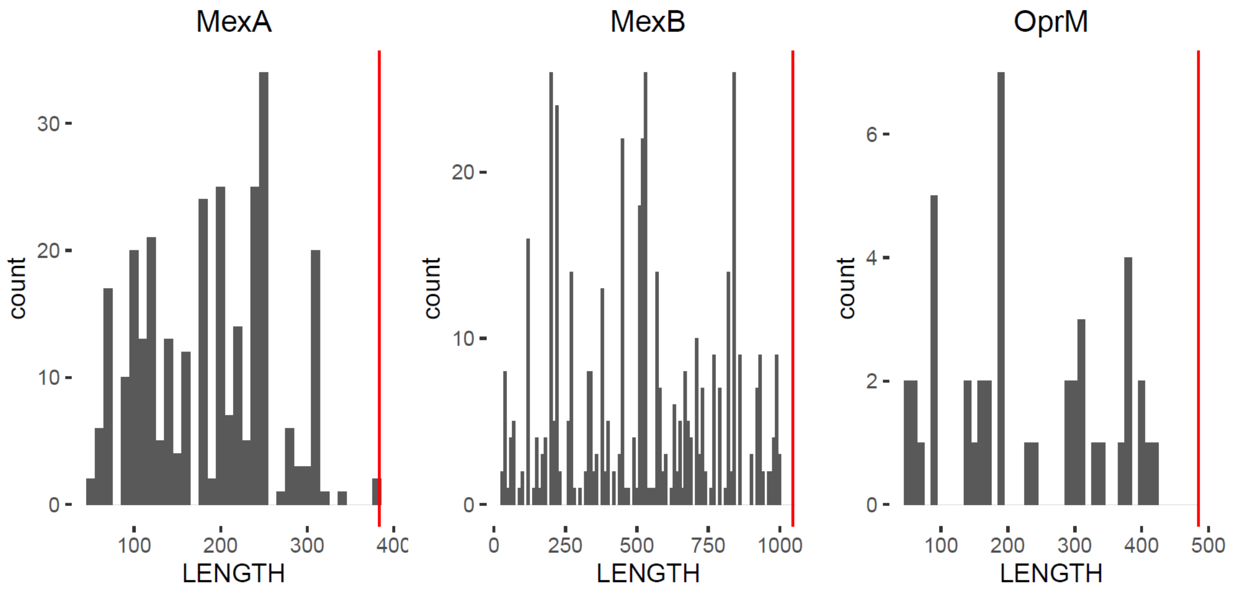

We next analyzed the sequence variants identified in the coding regions of *mexB*. We specifically focused on the MexB W753R mutation, 19 mutations from the literature which have been experimentally demonstrated to inactivate MexB[3-7] (Table RR5), and the distribution of other likely inactivating variants including stop codons and frameshifts.

Table RR5. Experimentally-validated MexB inactivating variants. "Mutations in the target codon" indicates the number of assemblies in the database found to have a sequence variant at the same amino acid position, while "Identical mutation" indicates whether or not the variant(s) corresponds identically to the mutation in the left-most column.

MexB mutation	Mutations in the target codon	Identical mutations	Reference
G220S	0	0	Nehme, Dominic, et al<br>Middlemiss, Jocelyn, K et al
T578I	1	1	Middlemiss, Jocelyn, K et al<br>Nehme, Dominic et al
T60I	2	0	Middlemiss, Jocelyn, K et al
S183L	2	2	Middlemiss, Jocelyn, K et al
M395I	0	0	Middlemiss, Jocelyn, K et al
A618T	0	0	Middlemiss, Jocelyn, K et al
R716H	2	0	Middlemiss, Jocelyn, K et al
S450L	1	0	Middlemiss, Jocelyn, K et al
E946K	0	0	Middlemiss, Jocelyn, K et al
C966Y	0	0	Middlemiss, Jocelyn, K et al
G51D	0	0	Middlemiss, Jocelyn, K et al
G754D	0	0	Middlemiss, Jocelyn, K et al
S977F	1	0	Middlemiss, Jocelyn, K et al
S462F	1	0	Middlemiss, Jocelyn, K et al
V928M	0	0	Middlemiss, Jocelyn, K et al
G220S	2	0	Middlemiss, Jocelyn, K et al
T578I	0	0	Middlemiss, Jocelyn, K et al
E864K	0	0	Middlemiss, Jocelyn, K et al
G1002D	0	0	Middlemiss, Jocelyn, K et al
D407N	9	0	Middlemiss, Jocelyn, K et al<br>Guan, Lan, et al
D408N	0	0	Middlemiss, Jocelyn, K et al<br>Guan, Lan, et al
R34A/D	0	0	Tsutsumi, Kenta, et al
R27A/D	0	0	Tsutsumi, Kenta, et al
T233A/V	0	0	Tsutsumi, Kenta, et al

We identified the MexB W753R mutation in 2/6820 queried genomes. But more broadly, we observed 462 potentially disruptive, non-synonymous variants among these MexB sequences, including 122 indels (106 frameshifting), 42 stop gained/start lost variants, 7 missense variants at positions experimentally validated to inactivate MexB, and two proven inactivating missense variants. (Table RR5). Putting together frameshifting indels and/or stop gained/start lost variants, variants in validated positions, as well as MexB W753R, these likely inactivating sequence

variants were present in 324/6820 genomes (4.8% of total). In order to adjust for clonality in this subset of assemblies, we conservatively assumed each unique high-impact variant (n=155) to represent a single lineage, obtaining a lower bound estimate of (2.2)%.

The more relevant calculation is what percentage of genomes have inactivation of the MexAB-OprM complex, not just MexB. To calculate this proportion, we additionally similarly identified disruptive variants in the coding sequences of MexA and OprM. We observed 277 and 232 nonsynonymous sequence variants in *mexA* and *oprM*, respectively, including 54 and 21 frameshifts, and 32 and 12 stop gained/start lost variants in *mexA* and *oprM*, respectively, distributed along coding sequences (Figure RR4, Table RR6).

Figure RR4. Distribution of stop gained/start lost/frameshifting variants along amino acid sequences of components of the MexAB-OprM efflux pump. The y-axis represents the aggregated sequence variant count, and the blue bar along the x-axis represents the full protein length as annotated in the PAO1 reference genome.

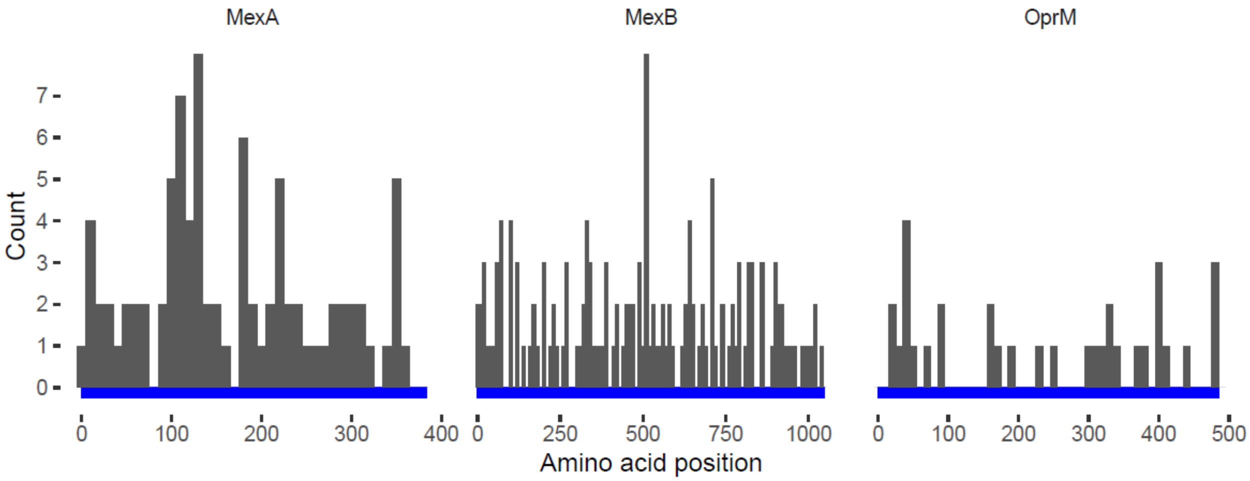

Table RR6. Variant characteristics found in MexAB-OprM protein sequences (n= 6820 assemblies).

Type	MexA	MexB	OprM	TOTAL
**Frameshift**	54	106	21	181
**Start lost / Stop gained**	32	42	12	86
**Missense**	177	298	194	669
**Conservative inframe insertion/deletion**	8	9	2	19
**Disruptive inframe insertion/deletion**	6	7	3	16
**Synonymous variant**	159	436	223	818
**TOTAL**	436	898	455	1789
**TOTAL NON-SYNONYMOUS**	277	462	232	971
**TOTAL HIGH IMPACT**	86	148	33	267

Together with the above, a total of 629/6820 (9.2%) genomes were found to have at least one sequence variant highly suggestive of an inactive MexAB-OprM efflux pump. To adjust for potential clonality in this subset, a conservative estimate assuming each unique variant to represent a clonal lineage was calculated, with a resulting total of 267/6820 (3.9%) of lineages are predicted to have an inactive MexAB-OprM efflux pump (Table RR7). We believe this percentage represents a lower bound, as we did not count the large number of uncharacterized nonsynonymous variants, many of which may alter function. A caveat as above is that we cannot definitively evaluate or control for potential sequencing or assembly errors or artifacts.

Table RR7: Highly disruptive variants in MexAB-OpM in the NCBI Pathogen Detection Database. Percentage calculations in the "Number of assemblies" column represent the number of assemblies containing variants in the given gene divided by the number of assemblies with at least one high-similarity BLASTp match for all proteins in the system (n = 6820). The percentage calculations in "Number of variants" column represent the number of unique variants divided by the same denominator above. This latter percentage represents a conservative estimate of the variant frequency adjusted for clonality with the assumption that all assemblies containing a given variant are clonal.

	**Number of variants (%)**	**Number of assemblies (%)**
**MexA**	86 (1.3)	199 (2.9)
**MexB**	148 (2.2)	335 (4.9)
**OprM**	33 (0.5)	124 (1.8)
**MexAB-OprM (total)**	267 (3.9)	629 (9.2)

We next asked how many assemblies in the database would be predicted to be both putative hypermutators and have a nonfunctional MexAB-OprM. Surprisingly, 91 out of 260 putative hypermutators (35%), were also found to have at least one sequence variant suggestive of an inactive MexAB-OprM, raising the possibility that hypermutation and MexAB-OprM inactivation may in fact occur frequently together. Once again, in order to adjust for clonality in this subset of assemblies, we conservatively assumed each individual disruptive variant (n = 47) to represent a single clonal lineage, obtaining a lower bound estimate of 47/260 (18%). These represent ~1.3% of all assemblies analyzed. This analysis suggests that concurrent hypermutator phenotypes and MexAB-OprM efflux pump disruption co-exist in a small, but easily detectable, proportion of the clinically relevant *P. aeruginosa* population.

We have added this information to the manuscripts as Supplemental Table 10 and have included additional text in lines 446-480, 634-638, and 1016-1030 (tracked version).

3. *The authors state in the discussion "Given these findings, identification of hypermutator phenotypes in the clinical microbiology lab, either through direct phenotypic testing or through targeted gene amplification and sequencing, may in the future inform optimized treatment decisions based on these differences in behavior." This is an important point. However, I think it needs to be put in the context of the percentage of time that hypermutators are differentially resistant to CZA. Did the authors happen to look back at*

the public data (in addition to the question in #1 above) and cross reference with any available AST data for CZA in those sequenced strains to determine how often CZA resistance is found (there might not be much out there)? This will be critical to making the point that knowing mutS status for example could translate into different clinical decisions.

Response: We agree with this reviewer that this is a key question. As we found above, a conservative estimate taking only indel/frameshifting/stop codon creation mutations into account suggests that at least 3.8% of *P. aeruginosa* isolates in the NCBI pathogen database are hypermutators (2.3% with the most conservative assumptions about redundancy), so it is fully expected that hypermutators occur commonly in clinical practice and that these isolates will experience selection under CZA, C/T, and cefepime. Ideally, one would like to have correlative phenotype-genotype data with antimicrobial susceptibility testing for these agents. Unfortunately, there is very scarce data on CZA susceptibility in public genomic databases as the reviewer suggests. Out of 7492 isolates in the NCBI Pathogen Detection database, only a small minority (149 genomes, 2%) had any antimicrobial susceptibility data, with 27 (0.3%) having CZA susceptibility data available. Only 7 out of the 260 putative hypermutator isolates as defined above had AST phenotypic data available, and only 1/5 had CZA data (CZA R). Interestingly, this one assembly did contain amino acid substitutions in the AmpC associated with ceftolozane/tazobactam resistance (PDC T105A and G391A)[8]. However, this is unfortunately just not enough data to draw any conclusions. This has been added in lines 481-488 (tracked numbering).

Importantly, we want to clarify we do not necessarily expect to see a <u>specific correlation between hypermutator phenotypes and CZA resistance.</u> More broadly, we would note that the literature suggests that hypermutation may accelerate the development of resistance to other categories of antibiotics in *P. aeruginosa* as well, though in our study we focused only on the development of resistance in response to CZA selection. Thus, the potential clinical relevance of determining the presence of a hypermutator phenotypes may extend more broadly to other antibiotic classes, but this was not addressed in our study.

In response to the reviewer's comment, we have added qualifying language to the discussion to clarify the limitations on the data supporting our suggestion in line xxx.

4. *What if anything is known about the existing transposon mutants for MexV and MexW in the same collection that was used for this study? Were they tested to see if the MIC to CZA changes and are the transposon insertions covering the same mutations that were found in the evolution experiments? The real point of this question is to get at whether the mutations that were found in MexV and MexW are the critical bases to determine a phenotype or whether a variety of mutations in these genes could lead to a similar phenotype.*

Response: We thank the reviewer for these important questions. The transposon mutants available in the University of Washington (Manoil lab) MPAO1 transposon collection are

predicted to cause inactivation of the genes targeted as they result in the incorporation of a large transposon at the integration site in the middle of coding sequence. In our study, we observed one mutation in the intergenic (non-coding) region upstream of the *mexV*/*mexW* operon that we believe increases expression of both genes based on RNA-seq data, and a second mutation in the coding sequence of the *mexW* gene resulting in a E36K substitution in the encoded MexW protein. Thus, while we would predict that transposon insertion mutants in MexV and MexW would inactivate the MexVW efflux pump, we believe the two mutations we observe to be "gain-of-function" mutations which increase expression and/or activity (e.g., altered transport specificity) of the MexVW efflux pump against CAZ, FEP, and C/T.

In response to the reviewer's question and to verify that we are indeed looking at gain-of-function mutations rather than loss-of-function mutations acting through some complex secondary mechanism to provide resistance, we evaluated the effects on antimicrobial resistance of both the *mexV* and *mexW* transposon insertion mutants from University of Washington and a constructed in-frame deletion of the MexVW. Susceptibility data for the *mexV* and *mexW* transposon insertion mutants are shown in Table RR8.

Table RR8. Susceptibility data for University of Washington *mexV* and *mexW* transposon insertion mutants. All MICs are given in μg/mL.

UW STRAIN NAME	PA ORF	GENE	CZA	CAZ
**MPAO1-WT**	-	-	0.75	0.75
**PW8386**	PA4374	*mexV*	0.75	0.5
**PW8385**	PA4374	*mexV*	0.75	0.5
**PW8390**	PA4375	*mexW*	0.75	0.5
**PW8389**	PA4375	*mexW*	0.75	0.5

To construct the *mexV*/*mexW* deletion mutant, we used a two-step allelic exchange approach (using the same approach as described in the Methods section of the main manuscript) to engineer a clean in-frame deletion of both *mexV* and *mexW* into an MPAO1-WT background, to generate strain MPAO1-Δ*mexV*Δ*mexW*. The susceptibility profiles of these mutants for CZA, CAZ, FEP and C/T are shown in the Table RR9.

Table RR9: Susceptibility profile of MPAO1-WT (n=2) compared to MPAO1-Δ*mexV*Δ*mexW* (n=4). CZA = Ceftazidime-avibactam; CAZ = Ceftazidime; FEP = Cefepime; C/T = Ceftolozane/tazobactam. All MICs are given in μg/mL.

STRAIN NAME	Replicate	CZA	CAZ	FEP	C/T
**MPAO1-WT**	1	0.875	0.75	0.75	0.38
**MPAO1-WT**	2	0.875	0.75	0.75	0.38
**MPAO1-ΔmexVΔmexW**	1	0.5	0.5	0.75	0.38
**MPAO1-ΔmexVΔmexW**	2	0.5	0.5	0.75	0.38
**MPAO1-ΔmexVΔmexW**	3	0.5	0.75	1.0	0.38
**MPAO1-ΔmexVΔmexW**	4	0.5	0.5	0.75	0.38

These results show that the two mutations found and tested in our study, the mutation upstream of the *mexV/mexW* operon and the MexW E36K mutation are gain-of-function mutations, not trivial loss-of-function mutations that are acting to provide resistance through some uncharacterized secondary mechanism.

In regard to the specific residues affected by the mutations described in our work, little is known about MexVW structure and function. We are currently working on elucidating additional functional details about this pump, but this work will take time to complete is beyond the scope of this manuscript.

<u>Methods used for responses to Reviewer #1:</u>

Assessment of putative hypermutators in NCBI Pathogen Detection Database: We used command line BLASTp v2.13.0 using as query the amino-acid sequences of *P. aeruginosa* PAO1 MutS (NP_252310.1), MutL (NP_253633.1), UvrD (NP_254130.1), MutT (NP_253090.1), MutY (NP_253834.1) and MutM (NP_249048.1) proteins against the *P. aeruginosa* assemblies in the NCBI Pathogen database (accessed 5/14/2022, n=6877, genomes with available assemblies and metadata). We retrieved all matches with e-value $<10^{-4}$ and percentage identity >94%; and counted the number of blast hits per assembly to evaluate potential gene duplications. Hit lengths were examined for assemblies with >1 BLASTp hit, and assemblies with hit lengths > 95% of the intact protein in PAO1 were assessed individually for putative duplication. Three assemblies were found to have potential duplications for: UvrD (GCA_011466835.1), MutY (GCA_013114955.1), and MutM (GCA_001180385.1); and were excluded from analysis. The remaining assemblies with >1 hit per query protein were explained by frameshifts and missense mutations resulting in more than one ORF annotated by prokka in homologous sequences and split annotation. For the next part of the analysis, we included assemblies with BLASTp hits for all these six proteins (n = 6805). These assemblies were used as input for snippy v4.4.1. with --contigs option to generate synthetic reads which were aligned back to the *P. aeruginosa* PAO1 genome (NC_002516.2) to generate variant calls. Variants were annotated with SnpEff v5.1d. The variants within the reading frames of *mutS*, *mutL*, *uvrD, mutT, mutY* or *mutM* were analyzed with the tidyverse Bioconductor package in R 4.13.

Assessment of putative MexAB-OprM inactivation variants in NCBI Pathogen Detection Database
Similar as above, we used a BLASTp approach with the PAO1 MexA (NP_249116.1), MexB (NP_249117.1) and OprM (NP_249118.1) sequences in order to identify possible assemblies with duplications or no identifiable homologues, using the same search cutoffs. We identified one assembly (GCA_000520315.1) containing a MexA duplication, which was excluded from analysis. Assemblies with at least one BLASTp hit for MexA, MexB and OprM (n=6820) were used as input for snippy and SnpEff to perform variant calling and annotation as described above.

Reviewer #2 (Remarks to the Author)
The manuscript explores mutational paths to betalactam (ceftazidime/avibactam) resistance in a hypermutating MMR-deficient Pseudomonas aeruginosa strain and compares them to paths

taken by non-mutator strains. They find that the hypermutating strain can attain much higher resistance than the wild-type and does so largely through unexpected mechanisms. Specifically, it seems that an initial random inactivating mutation of the most relevant efflux pump (MexAB) drove the hypermutating populations to alternative resistance paths, where the resistance can at least partly be attributed to an upregulation of an alternative, and less-known, MexVW efflux pump. The authors then confirm that the resistance paths through MexAB explain the resistance evolved in the wild-type and that MexVW gives a comparable advantage through an independent mechanism. The mansucript is written clearly and meaningfully combines experimental evolution, sequencing, transcriptional profiling and comparisons to deposited sequences to understand the evolutionary potential of Pseudomonas aeruginosa hypermutators.

Response: We sincerely appreciate this reviewer's highly insightful and greatly detailed review of our manuscript and positive summary of our work above.

However, some of the claims need more quantitative statistical support. Also, it is not clear how well these findings can generalize to other situations, since the most important finding comes down to a mutation which may have been anecdotal.

Response: We appreciate the general and specific concerns this reviewer raises, and we address them in the detailed response below.

General comments:

- There is some confusion as to how much of the extremely successful adaptation of the mutator is due to the fact that the mutation rate is higher and therefore the populations are not limited in time by waiting for the next mutation occurring, and how much is thanks to the fact that non-beneficial mutations can easily fix in mutators, opening doors to very different, and potentially more successful, adaptation paths. It seems the interpretations go exclusively in the direction of the second, which is more interesting, but I think some of the results could be explained with only the first.

Response: We thank the reviewer for articulating this very important clarifying point which has helped us to refine our arguments in the manuscript (specific responses below). We agree fully with the reviewer's general point that the experiments and genomic characterization we performed in this work, while extensive, do not allow us to distinguish the relative degrees to which (1) simple elevated mutation rate and (2) the ease with which non-beneficial mutations (including inactivating mutations in AMR genes) can fix (and thus change available evolutionary alternatives) contributed to the specific evolution of resistance we observed in this work, and more generally to what can be expected "in the wild" among clinical isolates. It may indeed be the case that elevated mutation rate may be sufficient to explain the results, and we have added clarifying language in lines 625-629 (tracked version).

To be more specific, would the mexVW mutations evolve in also the wildtype if given enough time, since they carry a benefit also without the deactivating mexB mutation (Supp. Fig. 9)? And if the mexB mutation is indeed important, is it sufficient? If any mexB loss of function mutation occurred on a non-mutator background, would it stimulate such quick adaptation by itself? Even

speculations that would answer these questions from the authors would be appreciated, if experiments seem outside of the scope.

<u>Response</u>: We agree that these are two pivotal questions. We are unable to perform additional *in vitro* evolution experiments to address these questions in the time frame that would be reasonable for this manuscript due to the regulatory approvals required for these types of experiments at the NIH, the time the actual experiments take (wild type mutation rates are low enough to require 20-30 daily passages of many parallel lineages to obtain adequate numbers of mutations and generation of resistance), and then genomic sequencing and analysis of the required hundreds of genomes is a major undertaking. However, there is some work in the literature that is helpful for answering these questions.

In the published literature, pathways to resistance involving alternative efflux pumps have been demonstrated in <u>non-hypermutator</u> *P. aeruginosa* when major efflux pump were inactive, either knocked-out (work of Li et al[9]) or under MexAB-OprM pharmacologic efflux pump inhibition[10]. In the work published by Li et al. that we cite in our manuscript as reference 57 in lines 522-526 (tracked version), one main finding was that MexVW could emerge as an alternative pathway to resistance to several antibiotic classes in a non-hypermutator, efflux-deficient strain. This was in fact the first description of MexVW in the literature as we note in our manuscript. MexVW has largely been ignored (with a few important exceptions) in the almost two decades since publication of this first manuscript so there really just isn't much data to allow us to speculate on how frequently MexVW-mediated resistance mechanisms emerge in wild type isolates.

On the basis of the above work, we believe that functional loss of MexAB-OprM efflux activity could be "sufficient" to lead to the evolution of alternative resistance mechanisms (including MexVW) in the absence of hypermutation, but we did not test this in our work.

As indicated above, we have clarified in lines 625-629 (tracked version) the limitations in the interpretations we have applied to our results.

Another general worry is that the reported findings, although very interesting, seem anecdotal and difficult to generalize. Is there a reason to think that other truly independent starting cultures of this or a different mutator would also get the deactivating mexB mutation with any notable frequency?

<u>Response</u>: We agree that this is a critical point to address. We found above in our work for the response to Reviewer 1, that disruptive MexAB-OprM mutations (frameshift and stop codons) occurred with surprisingly high frequency among clinical isolates in the NCBI database, with a lower bound estimate of 3.9% genomes (after conservative adjustment for clonal isolates) carrying an apparent inactivating variant in either MexA, MexB or OprM, and this number does not take into account all of the nonsynonymous variants that are observed, some proportion of which likely also completely or partially impair function. Thus, inactivating mutations/deletions in MexAB-OprM that may drive the evolution of alternative resistance mechanisms may in fact be relatively common and not anecdotal. Second, our lower bound estimate for hypermutators within the NCBI database based on strongly disruptive frameshifts and missense mutations in DNA repair genes is approximately 2.3% distinct genomes (after conservative adjustment for

clonality). Again, this estimate does not take into account the many nonsynonymous mutations that were observed in the various MMR and BER proteins and some proportion of these may result in hypermutator phenotypes. So hypermutators are also not rare. We do not know the frequency with which AMR genes are inactivated due to mutations resulting from hypermutation, but the analysis in the response to Reviewer 1 above suggests that the probability of co-occurrence of specific MexAB-OprM inactivation and hypermutation is not negligible (18% of presumed hypermutators in the NCBI pathogen database contain likely inactivating variants in MexAB-OprM). Thus, we believe that the evolutionary dynamics we describe, where AMR gene inactivation, either pre-existing or due to hypermutation, forces hypermutators to rapidly explore alternative resistance pathways is likely more general than has been appreciated.

In this context we think it is important to note that in the majority of clinical and *in vitro* studies describing MexAB-OprM mutations associated with cephalosporin resistance, there is no functional corroboration of the effect of the variant through the construction of isogenic mutants or other methods. It is conceivable that in a subset of these studies, MexAB-OprM variants in antibiotic adapted strains may actually be inactivating, and the isolates may have developed resistance through less appreciated pathways due to the unavailability of MexAB-OprM. In fact, many of these studies explicitly and intentionally filter out mutations in genes not known to be involved in antimicrobial resistance and so would miss such non-classical mutations. In our study, if we had we only sequenced the terminal hypermutator CZA-resistant isolates and looked only at known AMR genes, excluding all other mutations as has been done in other studies, we might have concluded that the MexB W753R variant was in fact responsible for the CZA resistance. We thus think it is very possible that evolution of resistance through alternative mechanisms may occur more commonly than has been appreciated due to the assumptions and approaches to analysis that have been applied to many prior published studies.

We have added additional clarifying text to the manuscript in lines 446-480, 634-638, and 994-1014 (tracked version) to address the points raised above.

Would they then go on this same new mexVW resistance path? Are there any sequenced hypermutating clinical isolates that would confirm the relevance of this resistance path and its link to a mutator background?

Response: As we noted, we are unable to perform additional sets of adequately sized *in vitro* evolution experiments to address this specific question, but the results of Li et al[9] do suggest that MevVW may be selected in non-mutator backgrounds. As we noted, MexVW has been largely ignored for the past two decades and we are not aware of other publications that shed light on this question. Furthermore, deeper exploration of this question is hampered by approaches frequently used to analyze genomic adaptation experiments. Given that other published studies in which hypermutator *P. aeruginosa* isolates have been evolved under antibiotic selection, mutations in non-canonical AMR genes have commonly been filtered out, it seems possible that mutations resulting in recruitment of MexVW may well have been missed. As an example, when reviewing the supplementary information provided in the work by Cabot et al[11] and Gomis et al[12], we noticed several variants present in the MexW coding sequence which are not mentioned in the manuscript (MexW Y94C in a non-CAZ hypermutator lineage #2 in Cabot et al, as well as MexW Y281C, A443T and V444A in lineages IMI1, IMI3 and IMI/REL3 respectively in Gomis

et al). But further specific study would be required to reach any conclusions about the functional consequences of these mutations.

Some relatively important claims are not backed up by a figure and quantitative analysis:

- *Figure 2 does not highlight the data that supports the main statement: mutators have mutations in genes unrelated to common antibiotic resistance mechanisms (e.g. betalactamases or efflux pumps). Instead of (or in addition to) the conservation and amino-acid similarity scores, the mutations should be labeled by whether they have or have not occurred in other published evolution experiments, and/or whether these genes are associated with known resistance mechanisms (e.g. differentiating whether they are expected to confer resistance also for this particular antibiotic or to others).*

Response: We thank the reviewer for highlighting this point. In response, we have performed an extensive literature search of all of the identified mutational targets of nonsynonymous variants in the hypermutator lineages and have now highlighted Figure 2 all those genes for which there is (adequately strong) functional evidence in the literature for association with resistance to third generation cephalosporins. The genes we added are: PA1436 (*mexN* of MexMN efflux pump), *acsB*, *ftsL*, *clpA*, *argJ*, PA0478-PA0479, with nine total fixed variants identified across these genes. It should be noted that 6 out of 9 fixed variants in these targets emerged after the CZA MIC was at least 128μg/mL, and were not in play when the clinical breakpoint of 16 μg/mL was reached.

- *Supplementary table 4 - this data needs to be shown in a figure. The claim seems to be that the mutations in the lineages evolved in antibiotic were different to the ones without, but this is not clearly supported. It also needs to be clear how many separate lineages were passaged.*

Response: We thank the reviewer for these important points to improve clarity. We agree it is important to highlight genes that were mutated without antibiotic pressure to make a distinction with those displayed on Figure 2. These genes have now been highlighted in Figure 2 with a superscript Ψ symbol and described in the figure legend. The number of lineages passaged with and without antibiotic have been clarified in the Supplementary Table 4 legend. The five genes (*pdtA, amaB, PA2462, pilY1* and *rfaE*) that were independently mutated in both the CZA exposure experiment as well as the no-antibiotic control experiment are mentioned in the main text in the original version of the manuscript. These genes were unusually long (>10 kilobases). As these represent only five genes containing six fixed variants out of a total of 139 fixed variants in the hypermutator lineages, we believe this supports the statement that a mostly different set of targets were mutated with and without antibiotics.

Minor comments:

- *Not clear what was expected for figure 1b vs what was found. Either a clearer explanation of what to look for in this plot or a different general analysis, e.g. of gene ontology/function would be more informative.*

Response: We agree with the reviewer that a clearer explanation for Figure 1b would aid interpretation. The goal of the figure is to give the reader a sense of the distribution of fixed

variants found in this study in the larger context of *Pseudomonas* spp. coding sequences (CDS), along the axes of average sequence conservation and gene prevalence across reference genomes. We did not intend a comparison between an "expected" distribution and an actual distribution per se. One of the key messages of the figure is that the fixed mutations in this study did not cluster only in poorly conserved genes for instance, but were broadly distributed across CDS roughly resembling the distribution of all CDS. We understand this might be confusing, so we have added additional explanatory text to the manuscript, found in lines 134-137 (tracked version).

We had previously performed gene ontology classification of all mutated targets occurring in the hypermutators, but did not include these results because they were difficult to interpret (essentially the genes mapped to high levels spanning much of the hierarchy of ontology). We now have included the gene ontology enrichment analysis below in Figure RR5 and as Supplementary Figure 2 in the manuscript. Additional text is included in lines 137-139 in the manuscript (tracked version).

Figure RR5: Gene Ontology enrichment analysis of targets of fixed mutations in *P. aeruginosa* hypermutator lineages. Significantly enriched GO terms (One-tailed Fisher's exact test $p<0.05$) for each GO domain are displayed in order of decreasing p-value. The x-axis represents the Log_2 (observed/expected) ratio. CC = cellular component

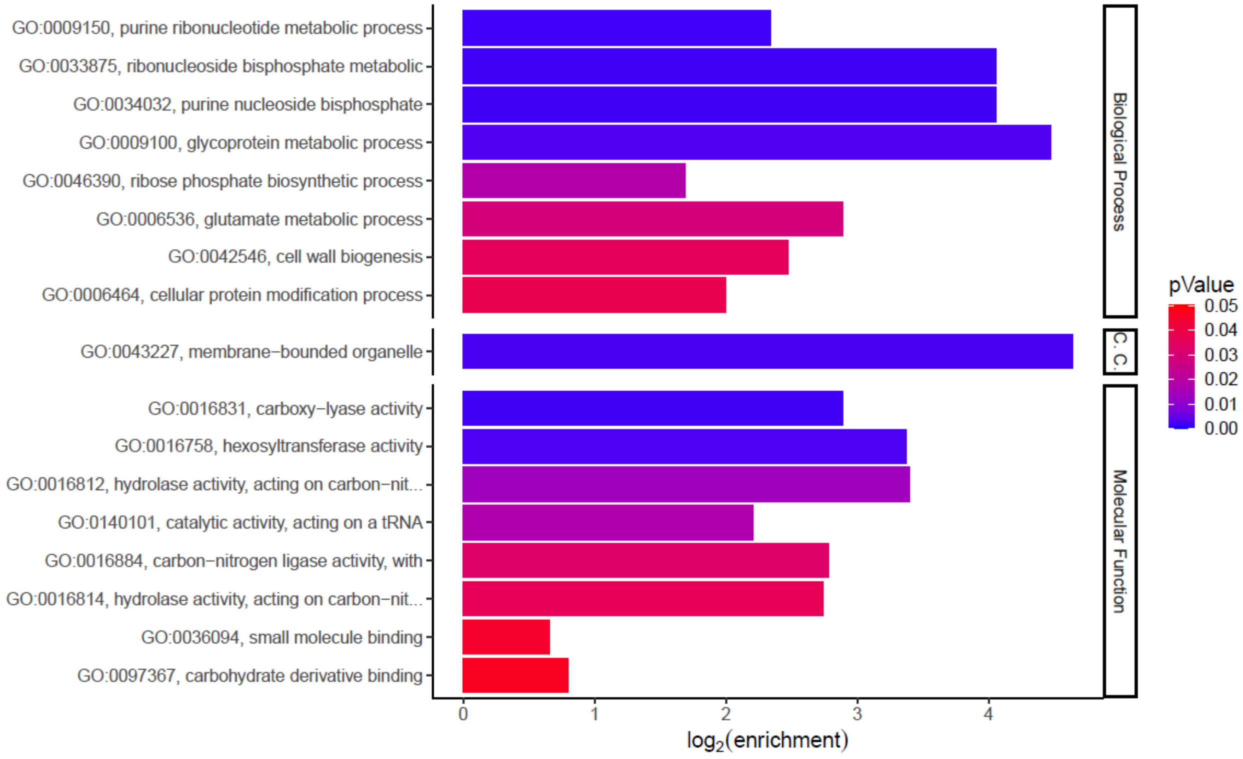

- *The information about the number of separate lineages of each background that were evolved is not clear enough. Especially Fig 1 calls for this type of information.*

Response: We thank the reviewer for this observation and appreciate the fact this information is difficult to distinguish in Figure 1. We have included the variant statistics for individual lineages as requested in Supplementary Figure 1.

- *Line 170. The important fraction here is not the number of mutations associated with resistance compared to all mutations that occurred in mutS. A much higher mutation rate will for sure yield many "hitchhiking" mutations which are not expected to have any benefit. The number of "relevant" targets in the mutator strain could potentially be compared to the number of fixed mutations in the wild type that underwent a similar increase in fold-resistance.*

Response: We appreciate the reviewer's point that the appropriate fraction is not the total number of mutations in hypermutators. We believe it would be challenging to determine "relevant" targets for both the WT and hypermutator lineages that would allow calculation of a accurate ratio as the reviewer suggests, due to complications in determining the set of genes under selection in these data. Thus, we have removed this fraction from the text and instead have listed explicitly the mutations that occur in targets that have been previously associated with third-generation cephalosporin resistance. We think this addresses the reviewer's point and concern without the added confusion of trying to justify a particular denominator and hope the reviewer concurs. The modified text is found in lines 207-212 (tracked version).

- *Line 196-197. I think there is a similar flaw in interpretation here. Shared mutations in mutator lineages do not suggest selective advantage if there is any chance of a common source of the mutation (which there clearly is here). High frequency of synonymous mutations, as shown in e.g. Fig 1a, support the fact that rate of hitchhiking was high and many fixed mutations are not expected to be beneficial. Only truly independently (ideally different loci) acquired mutations in the same gene targets could provide this type of support.*

<u>Response:</u> We certainly agree fully with the reviewer that shared mutations are most likely of common lineal origin here. We were specifically referring to mutations, which while common to multiple lineages, were originally not detected by isolate sequencing and therefore are present in a minority population in each lineage. Subsequently they became dominant in multiple independent lineages only after a few rounds of passaging under CZA selection. Since the variant frequency has clearly increased over rounds of selection, we believe that these mutations likely (though not definitively) provide fitness advantages. We have clarified and qualified the text with additional explanation in lines 251-253 (tracked version).

- *Line 292-293 I don't see why it would preclude direct comparison, the satellite colonies are expected with highly mutating strains, the zone where the lawn-forming bacteria are inhibited is the one to read out, after the recommended 16h this looks plausible to me. It*

would be very informative to add the MIC values of the evolved mutators to the plot in Supp. Figure 9.

Response: We agree with the reviewer that a readout of standardized susceptibility testing in the hypermutators would be useful for comparison to the same testing for engineered mutants, but we chose to exercise caution here as this interpretation requires that the outer halo actually represents the susceptibility level of background genome, while colonies within the zone of inhibition should represent spontaneous mutants able to tolerate higher antibiotic concentration. This is certainly a reasonable explanation (and the one usually chosen), but we have not done subculture and sequencing of the inner zone isolates to confirm the basis of this phenomenon systematically. There are other examples of similar phenomena that may represent poorly understood stochastic mechanisms induction in what surprisingly appear to be clonal populations on sequencing (for instance colistin resistance in *Enterobacteriales*[13]).

With the above caveats in mind, in Table RR10 (now also included in the manuscript as Supplementary Table 6) we now show the Kirby-Bauer 16-20 hr interpretation of the MPAO1-*mutS*^{Tn} ancestor and terminal MPAO1-*mutS*^{Tn} isolates for each of the three lineages (2X0, 2A12-1, 2B7-1 and 2D12-1), with the outer and inner halo diameters indicated when present. In order to determine the outer diameter, we visually identified the zone of confluent bacterial growth and measured the distance to the edge of the disk. We then calculated the inner diameter by measuring the distance between the disk edge and the closest satellite colony as prescribed in relevant CLSI documents. E-test results are shown for ceftolozane/tazobactam as an MIC (μg/mL).

Table RR10: Antimicrobial susceptibility testing of MPAO1-*mutS*^{Tn} ancestor and terminal isolates. Susceptibility testing in the hypermutators was complicated by the appearance of satellite colonies within the inner zones. We made the assumption that the outer zone reflected resistance conferred by the genomic background of the hypermutator and that the inner zone satellite colonies represented mutants able to resist higher concentrations of antibiotic. In order to determine the outer zone diameter, we visually identified the zone of confluent bacterial growth and measured the corresponding diameter. We then measured the inner diameter by measuring the distance between the disk edge and the closest satellite colony, and calculated the diameter using this distance. Diameters are shown in mm except for C/T, where testing was performed by Etest and results are show in mg/mL. Values within parenthesis correspond to the CLSI interpretation of the zone diameter. NA = no satellite colonies seen within zone. Letter in parenthesis indicates biological replicate and R1 and R2 represent technical replicates. Pip-Tazo = Piperacillin-Tazobactam.

	**MPAO1-*mutS*^{Tn} ancestor**		**Lineage 2A Passage 12**		**Lineage 2B Passage 7**		**Lineage 2D Passage 12**	
	**Outer**	**Inner**	**Outer**	**Inner**	**Outer**	**Inner**	**Outer**	**Inner**
**Pip-Tazo (A)**	29 (S)	NA	27 (S)	10 (R)	29 (S)	20 (I)	31 (S)	NA
**Pip-Tazo (B, R1)**	27 (S)	NA	26 (S)	12 (R)	31 (S)	16 (I)	29 (S)	NA
**Pip-Tazo (B, R2)**	27 (S)	NA	25 (S)	12 (R)	31 (S)	16 (I)	28 (S)	NA
**Meropenem (A)**	22 (S)	NA	25 (S)	15 (R)	28 (S)	18 (I)	29 (S)	18 (I)
**Meropenem (B, R1)**	30 (S)	NA	27 (S)	21 (S)	25 (S)	20 (S)	29 (S)	NA
**Meropenem (B, R2)**	31 (S)	NA	28 (S)	21 (S)	27 (S)	20 (S)	28 (S)	NA
**Imipenem (A)**	26 (S)	NA	19 (S)	NA	22 (S)	NA	24 (S)	NA

**Imipenem (B, R1)**	27 (S)	NA	21 (S)	NA	30 (S)	20 (S)	11 (R)	NA
**Imipenem (B, R2)**	27 (S)	NA	21 (S)	18 (I)	30 (S)	21 (S)	12 (R)	NA
**Aztreonam (A)**	24 (S)	NA	21 (S)	NA	20 (S)	NA	22 (S)	NA
**Aztreonam (B, R1)**	26 (S)	NA	17 (I)	NA	18 (I)	NA	13 (R)	NA
**Aztreonam (B, R2)**	-	-	17 (I)	NA	20 (I)	NA	13 (R)	NA
**Cefepime**	27 (S)	NA	6 (R)	NA	8 (R)	NA	6 (R)	NA
**C/T (E-test)**	1 (S)	NA	>256 (R)	NA	32 (R)	NA	64 (R)	NA

Methods used for responses to Reviewer #2:

Gene Ontology Enrichment Analysis. Among 139 fixed variants in MPAO1-$mutS^{Tn}$ lineages, we identified 78 distinct genes with nonsynonymous substitutions or indels. To perform functional annotation, the GO terms for the complete set of PAO1 loci were obtained from the Pseudomonas genome database[14]. A total of 4042 PAO1 GO annotated loci were suitable for analysis. We then utilized the topGO v2.46.0 R package[15] with the set of 78 targeted loci as input, using the weight01 algorithm to perform Fisher tests. The set of loci with a one-tailed Fisher's exact test $p<0.05$ are shown, with the Log_2 of the ratio of observed/expected hits displayed in the x-axis.

Reviewer #3: Comments to the authors

The manuscript by Dulanto et al. unravels the diverse evolutionary pathways that bacterial mutator clones can explore for the acquisition of antibiotic resistance in the opportunistic pathogen Pseudomonas aeruginosa. The authors perform in vitro adaptive evolution experiments by challenging reference PAO1 strain and a mutS isogenic mutant with increasing concentrations of ceftazidime-avibactam (CAZ). The experiments confirm that acquisition of CZA resistance is boosted by hypermutation compared with the wild-type non mutator strain. By applying whole-genome sequencing and transcriptomic of initial and final bacterial clones of the several mutator and wild-type lineages, the authors reveal a novel mechanism of mutation-driven β-lactam resistance based on the multidrug efflux pump MexVW, which has been previously described to confer resistance to antibiotics other than β-lactams. In contrast to wild-type lineages that are selected for mutations altering well-known CAZ resistance genes, including those leading to MexAB efflux pump overexpression, the work describes that MexVW-mediated resistance was acquired only by mutator lineages. Surprisingly, the authors find that the mutator inoculum hold an inactivating mutation in the mexB gene. Based upon this observation, they wonder if the MexVW resistance pathway evolved in mutators as an alternative mechanism, facing the primary mexB mutation that prevents the acquisition of resistance by MexAB efflux pump.

The contribution of this manuscript in P. aeruginosa resistance evolution processes associated with hypermutability is significant. The data provided support the conclusions. The manuscript is clear in communicating the main findings of the study.

Response: We greatly appreciate this reviewer's very thorough and thoughtful review of our manuscript and positive summary of our work above. We respond to each of the extremely useful comments below.

Major points
A major concern is respect to the size of the initial inoculum used for the experimental evolution assays. The smaller the initial inoculum, the smaller the probability to contain any preexisting mutant. This is particularly important when handling mutator strains and de novo mutations aim to be analyzed under the experimental conditions. To overcome this issue serial dilutions should be performed in order to obtain inocula ranging from 10 to 1000 cells. Here, authors have used a dilution of 0.05 OD600 (Line 502), which is a quite large inoculum that explains the heterogeneity of the initial population the authors observed.

Response: We agree with this reviewer that the critical population size in the initial inoculum is a very important variable in the experiment as it determines the amount of starting genetic diversity in the initial culture. Our choice of 0.05 OD600 corresponds to approximately 1.5×10^8 CFU/mL based on quantitative cell counts (see Methods section below). We initially attempted the experiments with much smaller inoculums but found a high rate of failures on passages into higher concentrations of antibiotic. We chose higher starting concentrations to maximize survival through aggressive selection steps. We understand that this involved a trade-off with choosing a larger starting critical population size and greater initial mutational diversity and, as a consequence, we observed multiple identical mutations in different lineages that most likely had a common origin in this initial starting mixture. We certainly do understand the reviewer's concern and attention to this important detail, and we spent a lot of time considering the pros and cons of the inoculum size we selected at the time we performed the experiments. But we believe that the main results of the study still stand independent of the starting inoculum, for instance the discovery of the contribution of MexVW to cephalosporin resistance. We would also point out that in the case of *in vivo* infection, the relevant critical population sizes on which antibiotic selection acts may be as large as 10^9 CFU or more in a bacterial abscess or in a bacterial pneumonia.

We have added information on the number of CFU calculated for our starting inoculum and explanation of choice in lines 669-671 (tracked version).

Still, in this case, one of these preexisting mutations in mutator lineages inoculum resulted to be mexB. As authors hypothesize, mexB background may lead to mutator lineages bias their evolution towards alternative resistance genetic pathway: the central message of the work. Although authors support this hypothesis determining that mexB preexisting mutation fully inactivates the main MexAB-mediated resistance mechanism, what would happen if evolution experiments were conducted with small mutator inoculums avoiding mexB mutations in the initial populations? Likewise, what would happen if the experimental evolution were conducted from a normomutator mexB mutant? These approaches could help further supporting the hypothesis

Response: We agree that these are very important questions and would be a very informative to address. However, we are unable to perform additional *in vitro* evolution experiments in the time frame that would be reasonable for this manuscript due to the regulatory approvals required for these types of experiments at the NIH, the time the actual experiments take (wild type mutation rates are low enough to require 20-30 daily passages of many parallel lineages to obtain adequate

numbers of mutations and generation of resistance), and then genomic sequencing and analysis of the required hundreds of genomes is a major undertaking. However, there is some work in the literature that is helpful for answering these questions as we reviewed in answering Reviewer 2 above and repeat here.

One of the first things we will restate from above is that likely inactivating variants (stop codons and frameshifting indels) in MexA, MexB and OprM in publicly available NCBI pathogen database genomes are surprisingly common at 3.9% (conservatively adjusted for clonality) and we believe this represents an approximate lower bound estimate (please see the calculations above in response to Reviewer 1). Thus, even though the MexB mutation was a chance occurrence in the experiments we performed, inactivating MexAB-OprM mutations appear to be much more common than appreciated and a proportionate amount of evolution of resistance is presumably taking place in this background, though surprisingly little attention has been paid to this fact in the *P. aeruginosa* genomics and AMR field.

As for what would happen if the hypermutator experiments *in vitro* evolution were repeated with limiting small initial population sizes to minimize mutational diversity, we do not know. Indeed, this would be a very informative series of experiments to do, and we are considering a variety of ways of tacking this problem, but it is unfortunately beyond the scope of what is possible for the current manuscript.

For normomutator WT with MexB inactivation, as we noted in response to Reviewer 2, the work published by Li et al. that we cite in our manuscript as reference 57, one main finding was that MexVW could emerge as an alternative pathway to resistance to several antibiotic classes in a non-hypermutator, efflux-deficient strain. (This was in fact the first description of MexVW in the literature as we note in our manuscript). Thus, normomutator isolates may as well follow alternative evolutionary pathways when the primary AMR genes are inactivated, including the specific discovery of MexVW-based mechanisms in the background of inactivated MexAB, though presumably will on average discover these resistance mutations more slowly than hypermutation operating on the same starting background.

Minor points
At least in the first paragraphs of the Introduction section that I could check in detail, it seems that many references do not specifically correspond with the stated text.
For instance:
-Lines 60-62: This sentence refers to hypermutation in chronic infection with P. aeruginosa that is underlain by the inactivation of genes involved in DNA repair.
Taddei 1997 and Yang 2008, could be replaced by some more accurate such as Oliver et al. (2000, Science 288: 1251-1254); Mandsberg et al. (2009, AAC 53: 2483-2491); Ciofu et al. (2005, AAC 49: 2276-2282).
I suggest the references be checked.

Response: We thank the reviewer for the very careful attention to the references, which we realize became misnumbered in several places within the text during the editing process, and we also appreciate that we may not have chosen the best references available. The references have

been double checked and updated where needed, and we have followed the reviewer's suggestions and added the above suggested references.

1. *Data and references related to the clinical PT strain are missing in Table S8.*

Response: We thank the reviewer for noting this oversight. References and description have now been added. The PT isolate is more extensively described in this group's prior publication: mBio. 2019;10(5). 10.1128/mBio.01822-19.

2. *Numbers in Line 113 do not match with those in Figure 1a.*

Response: We thank the review for the very careful review here. The numbers have been corrected in the figure.

3. *Is PDC deletion described in Table S3 (referred from line 124) the same that those described in lines 128-130). Anyway, details of PDC deletion in Table S3 should be included as well as the other indel included in the Table.*

Response: We thank the reviewer for raising this point. The PDC deletion in Supplementary Table 3 is the one described as referenced. This was a 21nt in-frame deletion resulting in a 7aa deletion in the PDC Ω-loop (238-244del) which has now been added to Supplementary Table 3. More detailed annotation of ClpA and CzcS indels have been added to Supplementary Table 3 as well.

4. *Table S3 only includes data from PT strain lineages. It could be quite informative the detail of all mutations occurred in genes related to β-lactam resistance in all analyzed lineages (MPAO1 wt, MPAO1 mutS and PT)*

Response: We thank the reviewer for this suggestion. Supplementary Table 3 displays the same information as in Figure 2, but for the PT lineages only. We chose not to include the variants acquired by the PT lineage in Figure 2 for two reasons: (1) variant calling analysis was done against a different, multi-contig reference genome, as described in Khil et al. (mBio 2019 Sep 17;10(5)), which was multi drug-resistant at baseline, and (2) most variants in MPAO1-*mutS*^{Tn} lineages have not been described in association with β-lactam resistance, while other important variants (such as the MexVW associated variants discussed) have not been traditionally considered β-lactam resistance genes and would not be shown. Thus, a table displaying only β-lactam resistance mutations might not accurately reflect the message the study is trying to convey.

Methods used for responses to Reviewer #3:

Estimation of CFU corresponding to 0.05 OD_{600} for MPAO1-WT. SBA plates were inoculated from frozen stock and incubated overnight at 37°C. Triplicate cell suspensions in LB broth were prepared by collecting colonies from these plates, titrated to OD600 = ~0.5 with a BioTek cytation5 instrument. The suspensions were further diluted 10x to achieve a measured

OD600 of 0.052, 0.056 and 0.054 for each replicate, respectively. A tenfold serial dilution (10^{-1} to 10^{-10}) was performed by serially adding 100 μL of cell suspension to 900 μL LB broth. LB agar plates were inoculated with 50 μL of each cell suspension and incubated overnight. The 10^{-5} plates had a colony count of 55, 81and 86 colonies respectively, which were extrapolated to 1.1, 1.6 and 1.7×10^{8} CFU/mL (average = 1.47×10^{8} CFU/mL) in the OD600 = 0.05 starting suspension.

References

1 snippy: fast bacterial variant calling from NGS reads (https://github.com/tseemann/snippy, 2015).

2 Cingolani, P. *et al.* A program for annotating and predicting the effects of single nucleotide polymorphisms, SnpEff: SNPs in the genome of Drosophila melanogaster strain w1118; iso-2; iso-3. *Fly (Austin)* **6**, 80-92, doi:10.4161/fly.19695 (2012).

3 Guan, L. & Nakae, T. Identification of essential charged residues in transmembrane segments of the multidrug transporter MexB of Pseudomonas aeruginosa. *J Bacteriol* **183**, 1734-1739, doi:10.1128/JB.183.5.1734-1739.2001 (2001).

4 Middlemiss, J. K. & Poole, K. Differential impact of MexB mutations on substrate selectivity of the MexAB-OprM multidrug efflux pump of Pseudomonas aeruginosa. *J Bacteriol* **186**, 1258-1269, doi:10.1128/JB.186.5.1258-1269.2004 (2004).

5 Nehme, D. & Poole, K. Interaction of the MexA and MexB components of the MexAB-OprM multidrug efflux system of Pseudomonas aeruginosa: identification of MexA extragenic suppressors of a T578I mutation in MexB. *Antimicrob Agents Chemother* **49**, 4375-4378, doi:10.1128/AAC.49.10.4375-4378.2005 (2005).

6 Nehme, D. & Poole, K. Assembly of the MexAB-OprM multidrug pump of Pseudomonas aeruginosa: component interactions defined by the study of pump mutant suppressors. *J Bacteriol* **189**, 6118-6127, doi:10.1128/JB.00718-07 (2007).

7 Tsutsumi, K. *et al.* Structures of the wild-type MexAB-OprM tripartite pump reveal its complex formation and drug efflux mechanism. *Nat Commun* **10**, 1520, doi:10.1038/s41467-019-09463-9 (2019).

8 Rubio, A. M. *et al.* In Vitro Susceptibility of Multidrug-Resistant Pseudomonas aeruginosa following Treatment-Emergent Resistance to Ceftolozane-Tazobactam. *Antimicrob Agents Chemother* **65**, doi:10.1128/AAC.00084-21 (2021).

9 Li, Y. *et al.* A new member of the tripartite multidrug efflux pumps, MexVW-OprM, in Pseudomonas aeruginosa. *J Antimicrob Chemother* **52**, 572-575, doi:10.1093/jac/dkg390 (2003).

10 Ranjitkar, S. *et al.* Target (MexB)- and Efflux-Based Mechanisms Decreasing the Effectiveness of the Efflux Pump Inhibitor D13-9001 in Pseudomonas aeruginosa PAO1: Uncovering a New Role for MexMN-OprM in Efflux of beta-Lactams and a Novel Regulatory Circuit (MmnRS) Controlling MexMN Expression. *Antimicrob Agents Chemother* **63**, doi:10.1128/AAC.01718-18 (2019).

11 Cabot, G. *et al.* Evolution of Pseudomonas aeruginosa Antimicrobial Resistance and Fitness under Low and High Mutation Rates. *Antimicrob Agents Chemother* **60**, 1767-1778, doi:10.1128/AAC.02676-15 (2016).

12 Gomis-Font, M. A. *et al.* In vitro dynamics and mechanisms of resistance development to imipenem and imipenem/relebactam in Pseudomonas aeruginosa. *J Antimicrob Chemother* **75**, 2508-2515, doi:10.1093/jac/dkaa206 (2020).
13 Band, V. I. *et al.* Carbapenem-Resistant Klebsiella pneumoniae Exhibiting Clinically Undetected Colistin Heteroresistance Leads to Treatment Failure in a Murine Model of Infection. *mBio* **9**, doi:10.1128/mBio.02448-17 (2018).
14 Winsor, G. L. *et al.* Enhanced annotations and features for comparing thousands of Pseudomonas genomes in the Pseudomonas genome database. *Nucleic Acids Res* **44**, D646-653, doi:10.1093/nar/gkv1227 (2016).
15 topGO: Enrichment Analysis for Gene Ontology. R package version 2.48.0 (2022).
