# Supplementary material for: Hypermutator strains of Pseudomonas aeruginosa reveal novel pathways of resistance to combinations of cephalosporin antibiotics and beta-lactamase inhibitors
Source: PLoS Biol. 2022 Nov 18;20(11):e3001878. doi: 10.1371/journal.pbio.3001878 (PMC9718400; doi:10.1371/journal.pbio.3001878)
Supplement: Attachment — Submitted filename: RTR2.pdf [file pbio.3001878.s031.pdf]

## Response to Reviewers

**<u>Original title</u>**: Novel Mechanisms of Resistance to Ceftazidime/Avibactam and Ceftolozane/Tazobactam in Mismatch Repair-Deficient *Pseudomonas aeruginosa*

**<u>New title:</u>** Hypermutator strains of *Pseudomonas aeruginosa* reveal novel pathways of resistance to combinations of cephalosporin antibiotics and beta-lactamase inhibitors

Dulanto Chiang A, Patil PP, Beka L, Youn JH, Launay A, Bonomo RA, Khil PP, Dekker JP

Manuscript # PBIOLOGY-D-22-00962R2

**Editor's comments:**

*Based on the reviews, we are likely to accept this manuscript for publication, provided you satisfactorily address the remaining points raised by the reviewers. In particular, reviewer #2 wants you to add some more argumentation about why the mutations found in the hypermutating strain are beneficial. This reviewer also suggests to include in the manuscript your response to the comment about mexB deactivation frequency. Please also make sure to address the following data and other policy-related requests.*

**<u>Response:</u>** We are delighted that this manuscript is likely to be accepted for publication. We sincerely thank the three reviewers and the Editor again for the extremely thorough and thoughtful reviews of our manuscript and further comments. These additional suggestions have further improved the manuscript, and we are very appreciative of the significant effort and time to critique our work. Below we answer each of the additional questions raised, both editorial and those of reviewer 2.

*1. We recommend a change in the title: "Hypermutator strains of Pseudomonas aeruginosa reveal novel pathways of resistance to combinations of cephalosporin antibiotics and beta-lactamase inhibitors ". This is a suggestion, so please modify as you think it fits better.*

**<u>Response:</u>** We appreciate the suggestion for modification of the title and concur with the change. The original title was chosen to be more specific in naming both the mechanism of hypermutation among the different varieties and the names of the specific antimicrobial combinations addressed. We agree that the more general title is still accurate and may attract more readers.

*2. DATA POLICY:*
*You may be aware of the PLOS Data Policy, which requires that all data be made available without restriction: http://journals.plos.org/plosbiology/s/data-availability. For more information, please also see this editorial: http://dx.doi.org/10.1371/journal.pbio.1001797*

*Note that we do not require all raw data. Rather, we ask that all individual quantitative observations that underlie the data summarized in the figures and results of your paper be made available in one of the following forms:*

*A) Supplementary files (e.g., excel). Please ensure that all data files are uploaded as 'Supporting Information' and are invariably referred to (in the manuscript, figure legends, and the Description field when uploading your files) using the following format verbatim: S1 Data, S2 Data, etc. Multiple panels of a single or even several figures can be included as multiple sheets in one excel file that is saved using exactly the following convention: S1_Data.xlsx (using an underscore).*

*B) Deposition in a publicly available repository. Please also provide the accession code or a reviewer link so that we may view your data before publication.*

*Regardless of the method selected, please ensure that you provide the individual numerical values that underlie the summary data displayed in the following figure panels as they are essential for readers to assess your analysis and to reproduce it: Figures 1AB, 2, 3ABC, 4ABC, and Supplementary Figures SF1, SF2, SF3, SF4, SF5, SF6, SF7, SF8, SF9, SF11.*

*NOTE: the numerical data provided should include all replicates AND the way in which the plotted mean and errors were derived (it should not present only the mean/average values).*

*Please also ensure that figure legends in your manuscript include information on where the underlying data can be found, and ensure your supplemental data file/s has a legend.*

*Please ensure that your Data Statement in the submission system accurately describes where your data can be found.*

**Response:** We have followed all of the instructions above. We have added a Supplementary file named S2 Data (S2_Data.xlsx) containing the underlying numerical data to recreate all main and supplementary figures. The remaining supplementary data files have been renamed accordingly to accommodate this file, as S1 Data and S3-S6 Data

**Reviewer #1 (Remarks to the Author)**

*The authors have responded to my comments and questions in a thorough manner. The additional analysis looking for the presence of some of these mutations in the public database is an additional strength to the manuscript.*

**Response:** We greatly appreciate this reviewer's positive evaluation of our responses to comments and again express gratitude for the thoughtful review and very helpful critiques the reviewer provided.

**Reviewer #2 (Remarks to the Author)**

*I appreciate the thought and work put into addressing my comments and indeed, all major points have been addressed. The most important was the literature search and analysis confirming that mutators, mexB deactivating mutations and likely even their combinations are common in*

*clinical strains. This does make it plausible that the scenario the authors find in the evolution experiments would be relevant for the clinic as well.*

**Response:** We thank this reviewer again for the very insightful and greatly detailed review of our manuscript and positive summary of our work and our response to comments above.

*Only very few minor points remain:*

*-In the now revised explanation of why the authors believe the mutations found in the hypermutating strain are indeed beneficial (starting line 211 in tracked version), there is still a flaw. Even the increase in frequency of a mutation under antibiotic selection cannot by itself suggest each such mutation's role in antibiotic resistance. It is clear that the mutator acquired some mutations that gave it resistance, but very likely the remaining ones (likely a majority) just happened to appear on the background of beneficial ones and that is the reason for their increase in frequency, not their effect on fitness. Specifically in hypermutating strains, this is extremely common. This is mainly a point to improve the argumentation. In principle, I agree with the authors that it is very likely that some of the mutations identified in the mutator strains are actually previously unidentified resistance mutations. It is very difficult to understand which ones, just from the experiments conducted, so this warrants further study, outside the scope of this manuscript.*

**Response:** We agree with this reviewer that it is likely that the majority of the mutations that occurred in the hypermutators are unrelated to antibiotic resistance and that many or most were co-selected because they occurred in the background of mutations that did improve fitness. It was not our intention to imply otherwise. We have added clarifying sentences to the text in lines 203-207 (tracked version) to emphasize this point.

*-In the response to my comment about mexB deactivation frequency, the authors argue that mexB mutations are likely to be wrongly classified as mexB activating/modifying and not deactivating as they confirm. I could not find this note in the updated version of the manuscript (somehow the line numbers did not match in the tracked file, so I am not 100% sure), but think is very interesting, so would be worth including if the space limitations allow.*

**Response:** We thank the reviewer for noting this omission and agree that this point should be included. We have added additional text in the manuscript in lines 613-618 (tracked version).

**Reviewer #3: Comments to the authors**

*The authors have improved the manuscript accordingly with the comments and suggestions expressed by the reviewers. I'm pleased with the changes included in the revised version of the article as well as with all new data, analyses and arguments they detaily addressed in the response letter.*

**Response:** We again express gratitude for this reviewer's very thorough and thoughtful review of our manuscript and positive summary of the comments above.
